# Supplementary material for: Identification of peripheral neural circuits that regulate heart rate using optogenetic and viral vector strategies
Source: Nat Commun. 2019 Apr 26;10:1944. doi: 10.1038/s41467-019-09770-1 (PMC6486614; doi:10.1038/s41467-019-09770-1)
Supplement: Supplementary file 1 — Supplementary Information [file 41467_2019_9770_MOESM1_ESM.pdf]

# Supplementary Information

# Identification of peripheral neural circuits that regulate heart rate using optogenetic and viral vector strategies

**Rajendran et al.**

| Power (mW) ($n$ = 6) | Baseline heart rate (bpm) | Stimulation heart rate (bpm) | Δ Heart rate (%) |
|---|---|---|---|
| 0 | 355.8 ± 37.7 | 355.1 ± 38.0 | -0.2 ± 0.2 |
| 38 | 365.8 ± 34.1 | 307.2 ± 46.1 | -16.0 ± 8.7 |
| 115 | 353.0 ± 35.4 | 228.3 ± 23.7 | -31.8 ± 11.0 |
| 226 | 379.2 ± 35.6 | 200.3 ± 30.0 | -45.9 ± 9.7 |
| **Frequency (Hz)** ($n$ = 6) | | | |
| 1 | 353.5 ± 27.2 | 324.6 ± 13.2 | -7.2 ± 3.3 |
| 5 | 357.8 ± 25.7 | 282.4 ± 18.3 | -19.7 ± 7.1 |
| 10 | 347.9 ± 23.0 | 219.7 ± 21.0 | -35.2 ± 8.0 |
| 20 | 351.2 ± 29.0 | 164.5 ± 42.9 | -50.0 ± 14.9 |
| **Pulse width (ms)** ($n$ = 6) | | | |
| 1 | 355.6 ± 28.5 | 278.1 ± 23.8 | -20.4 ± 8.0 |
| 2 | 362.1 ± 30.4 | 244.8 ± 25.4 | -29.7 ± 10.3 |
| 10 | 356.6 ± 25.5 | 227.1 ± 21.6 | -34.4 ± 8.2 |
| **Atropine** ($n$ = 5) | | | |
| Pre | 354.9 ± 35.3 | 224.2 ± 25.0 | -33.5 ± 11.0* |
| Post | 333.8 ± 37.7 | 333.0 ± 37.9 | -0.3 ± 0.2 |

**Supplementary Table 1. *Ex vivo* optogenetic stimulation of cholinergic neurons in the IPV-GP.** Table corresponds to Figure 3. Dose response curves summarizing the effects of altering light pulse power, frequency, and pulse width on heart rate. Summary of the heart rate response to stimulation before versus after atropine administration ($t_4$ = 2.993, *$P$ = 0.0402). mean ± s.e.m.; paired, two-tailed $t$-test.

| Frequency (Hz) (*n* = 8) | Intact RVNS | | | | | |
|---|---|---|---|---|---|---|
| | Optical | | | Electrical | | |
| | Baseline heart rate (bpm) | Stimulation heart rate (bpm) | Δ Heart rate (%) | Baseline heart rate (bpm) | Stimulation heart rate (bpm) | Δ Heart rate (%) |
| 1 | 560.7 ± 5.5 | 552.5 ± 5.9 | -1.5 ± 0.2 | 594.1 ± 4.4 | 587.9 ± 4.5 | -1.0 ± 0.2 |
| 5 | 561.6 ± 6.6 | 533.6 ± 5.3 | -5.0 ± 0.3 | 593.4 ± 5.5 | 556.0 ± 8.7 | -6.3 ± 0.8 |
| 10 | 552.0 ± 8.0 | 481.6 ± 8.7 | -12.8 ± 0.4 | 590.7 ± 6.1 | 509.5 ± 6.3 | -13.8 ± 0.6 |
| 20 | 558.3 ± 6.3 | 401.3 ± 13.0 | -28.1 ± 2.2 | 591.6 ± 5.7 | 370.3 ± 32.0 | -37.7 ± 5.0 |
| Frequency (Hz) (*n* = 3) | RVNx caudal RVNS | | | | | |
| 1 | 545.8 ± 12.9 | 535.9 ± 13.1 | -1.8 ± 0.6 | 564.2 ± 8.9 | 555.3 ± 8.0 | -1.6 ± 0.5 |
| 5 | 550.9 ± 11.5 | 501.5 ± 9.2 | -9.0 ± 1.0 | 567.7 ± 7.9 | 519.9 ± 13.3 | -8.4 ± 1.9 |
| 10 | 562.2 ± 8.2 | 459.6 ± 19.5 | -18.3 ± 2.5 | 568.2 ± 4.6 | 449.2 ± 33.0 | -21.0 ± 5.7 |
| 20 | 549.3 ± 11.5 | 302.9 ± 46.0 | -44.9 ± 8.1 | 564.2 ± 7.4 | 276.2 ± 47.6 | -51.0 ± 8.5 |
| Frequency (Hz) (*n* = 3) | BVNx caudal RVNS | | | | | |
| 1 | 530.0 ± 6.6 | 516.4 ± 5.9 | -2.6 ± 0.4 | 558.9 ± 7.1 | 548.3 ± 10.6 | -1.9 ± 0.7 |
| 5 | 534.0 ± 2.7 | 493.6 ± 5.7 | -7.5 ± 1.5 | 549.8 ± 10.6 | 508.3 ± 28.5 | -7.7 ± 3.4 |
| 10 | 560.6 ± 6.4 | 478.2 ± 16.7 | -14.7 ± 2.3 | 546.9 ± 9.5 | 457.2 ± 33.8 | -16.5 ± 5.1 |
| 20 | 545.6 ± 3.0 | 370.8 ± 44.5 | -32.0 ± 8.3 | 551.5 ± 8.1 | 354.4 ± 69.3 | -36.1 ± 11.5 |
| Frequency (Hz) (*n* = 5) | RVNx cranial RVNS | | | | | |
| 1 | 533.4 ± 10.6 | 531.2 ± 10.7 | -0.4 ± 0.1 | 569.5 ± 13.0 | 568.2 ± 13.5 | -0.2 ± 0.2 |
| 5 | 539.3 ± 11.0 | 536.9 ± 10.5 | -0.4 ± 0.2 | 569.6 ± 14.1 | 559.0 ± 19.5 | -2.0 ± 1.3 |
| 10 | 556.6 ± 12.9 | 556.0 ± 12.7 | -0.1 ± 0.1* | 576.1 ± 10.8 | 539.5 ± 18.8 | -6.5 ± 1.8* |
| 20 | 550.1 ± 12.1 | 549.3 ± 11.7 | -0.1 ± 0.1** | 576.1 ± 11.2 | 519.5 ± 17.3 | -9.9 ± 1.8** |
| Frequency (Hz) (*n* = 5) | BVNx cranial RVNS | | | | | |
| 1 | 518.5 ± 7.4 | 519.7 ± 8.5 | 0.2 ± 0.2 | 546.7 ± 7.5 | 546.9 ± 8.0 | 0.0 ± 0.2 |
| 5 | 526.1 ± 9.0 | 525.5 ± 9.4 | -0.1 ± 0.1 | 545.2 ± 9.1 | 545.9 ± 8.9 | 0.1 ± 0.1 |
| 10 | 547.4 ± 5.9 | 545.5 ± 5.5 | -0.3 ± 0.2 | 542.4 ± 8.5 | 539.7 ± 11.0 | -0.5 ± 0.5 |
| 20 | 535.7 ± 6.9 | 534.9 ± 7.2 | -0.2 ± 0.1** | 545.6 ± 7.8 | 528.3 ± 8.7 | -3.2 ± 0.4** |

**Supplementary Table 2. *In vivo* optogenetic versus electrical stimulation of the vagus nerve.** Table corresponds to Figure 4. Frequency response curves summarizing the effects of optogenetic versus electrical stimulation of the right vagus nerve on heart rate in the intact state, of the caudal end following RVNx and BVNx, and of the cranial end following RVNx ($t_4$ = 3.576, *$P$ = 0.0232 at 10 Hz; $t_4$ = 5.229, **$P$ = 0.0064 at 20 Hz) and BVNx ($t_4$ = 8.588, **$P$ = 0.0010 at 20 Hz). mean ± s.e.m.; paired, two-tailed *t*-test.

| Frequency (Hz) ($n$ = 7) | Baseline heart rate (bpm) | Stimulation heart rate (bpm) | Δ Heart rate (%) |
|---|---|---|---|
| 1 | 464.8 ± 22.6 | 471.7 ± 22.1 | 1.5 ± 0.4 |
| 2 | 458.6 ± 21.3 | 476.2 ± 22.6 | 3.8 ± 0.6 |
| 5 | 459.6 ± 22.1 | 486.5 ± 20.6 | 6.1 ± 1.1 |
| 10 | 455.7 ± 20.8 | 499.5 ± 20.4 | 9.9 ± 1.8 |
| 20 | 462.4 ± 20.2 | 499.8 ± 18.3 | 8.3 ± 1.1 |
| **Pulse width (ms)** ($n$ = 6) | | | |
| 1 | 469.5 ± 37.5 | 482.9 ± 33.8 | 3.4 ± 1.6 |
| 2 | 475.0 ± 38.8 | 492.0 ± 35.3 | 4.1 ± 1.8 |
| 5 | 475.3 ± 36.3 | 500.4 ± 33.0 | 5.9 ± 2.4 |
| 10 | 464.2 ± 32.0 | 499.0 ± 31.2 | 7.9 ± 2.2 |
| **RSG versus RT2G** ($n$ = 7) | | | |
| RSG | 461.7 ± 24.2 | 504.1 ± 23.1 | 9.5 ± 1.8** |
| RT2G | 486.1 ± 39.4 | 487.6 ± 39.3 | 0.3 ± 0.2 |
| **Propranolol** ($n$ = 4) | | | |
| Pre | 471.5 ± 23.4 | 498.0 ± 26.0 | 5.6 ± 1.4* |
| Post | 364.1 ± 20.1 | 364.7 ± 20.5 | 0.1 ± 0.1 |

**Supplementary Table 3. *In vivo* optogenetic stimulation of noradrenergic neurons in the RSG.** Table corresponds to Figure 6. Dose response curves summarizing the effects of altering frequency and pulse width on heart rate. Summary of the heart rate response to stimulation of the RSG versus RT2G ($t_6$ = 5.435, ***P* = 0.0016). Summary of the heart rate response to RSG stimulation before versus after propranolol administration ($t_3$ = 3.951, **P* = 0.0289). mean ± s.e.m.; paired, two-tailed *t*-test.

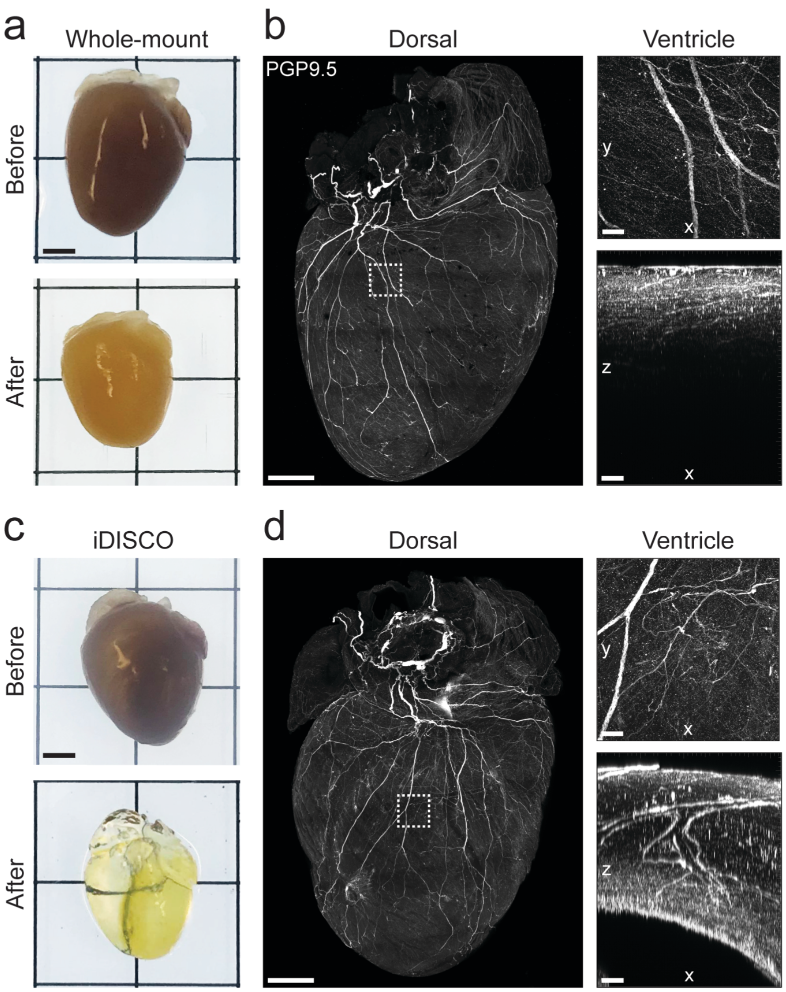


**Supplementary Figure 1. Whole-mount stained heart versus iDISCO-cleared heart.** (**a**) A heart before (top) and after (bottom) whole-mount staining. (**b**) 3D confocal projection of the dorsal side of a heart (2000 µm z-stack) whole-mount stained with PGP9.5 (gray). Insets show a MIP image of the left ventricular wall (top right) and a 1000 µm-thick 3D projection of the left ventricular wall (bottom right). (**c**) A whole heart (top) was rendered transparent (bottom) using the iDISCO protocol. (**d**) 3D confocal projection of the dorsal side of an iDISCO-cleared heart (2000 µm z-stack) stained with PGP9.5 (gray). Insets show a MIP image of the left ventricular wall (top right) and a 1000 µm-thick 3D projection of the left ventricular wall (bottom right). In contrast to whole-mount stained hearts, nerve fibers could be visualized throughout the entire thickness of myocardium in iDISCO-cleared hearts. Scale bars are 2 mm (**a**, **c**), 1 mm (**b** (left), **d** (left)), and 100 µm (**b** (right), **d** (right)).

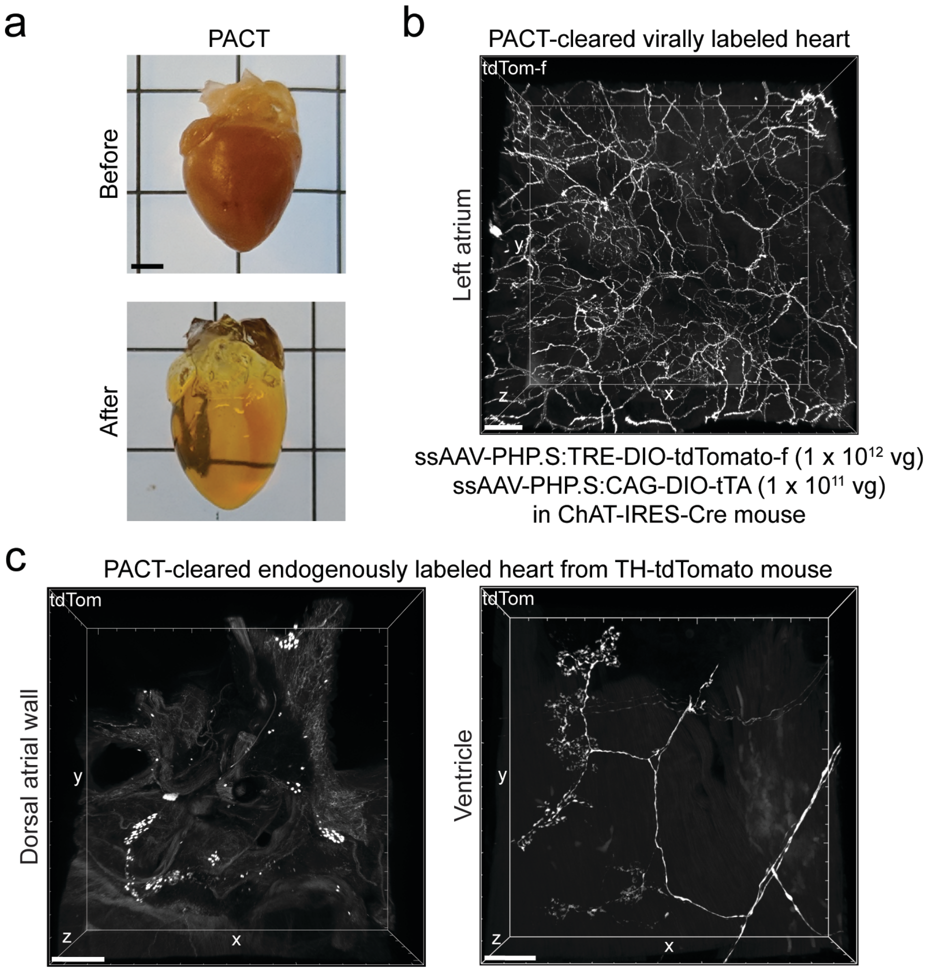


**Supplementary Figure 2. PACT clearing preserves fluorescence in virally and endogenously labeled hearts.** (**a**) Example of a whole heart (top) that was rendered transparent (bottom) using the PACT protocol. (**b**) ssAAV-PHP.S:TRE-DIO-tdTomato-farnesylated (1 x $10^{12}$ vg) and ssAAV-PHP.S:CAG-DIO-tTA (1 x $10^{11}$ vg) were systemically administered to a ChAT-IRES-Cre mouse. Four weeks later, the whole heart was cleared using the PACT protocol. A 402 µm-thick 3D projection of the left atrium showing cholinergic nerve fibers with native tdTomato fluorescence (gray). (**c**) tdTomato was expressed in noradrenergic neurons by crossing transgenic TH-IRES-Cre mice with Ai14 reporter mice containing a Cre-dependent tdTomato fluorescent protein allele (TH-tdTomato). The dorsal half of a heart from a TH-tdTomato mouse was cleared using the PACT protocol. A 1000 µm-thick 3D projection of the dorsal atrial wall showing noradrenergic neurons in cardiac ganglia with native tdTomato fluorescence (gray) (left). An 85 µm-thick 3D projection of the ventricle showing noradrenergic nerves and nerve terminals (right). Scale bar is 2 mm (**a**), 100 µm (**b**), 500 µm (**c**, left), and 50 µm (**c**, right).

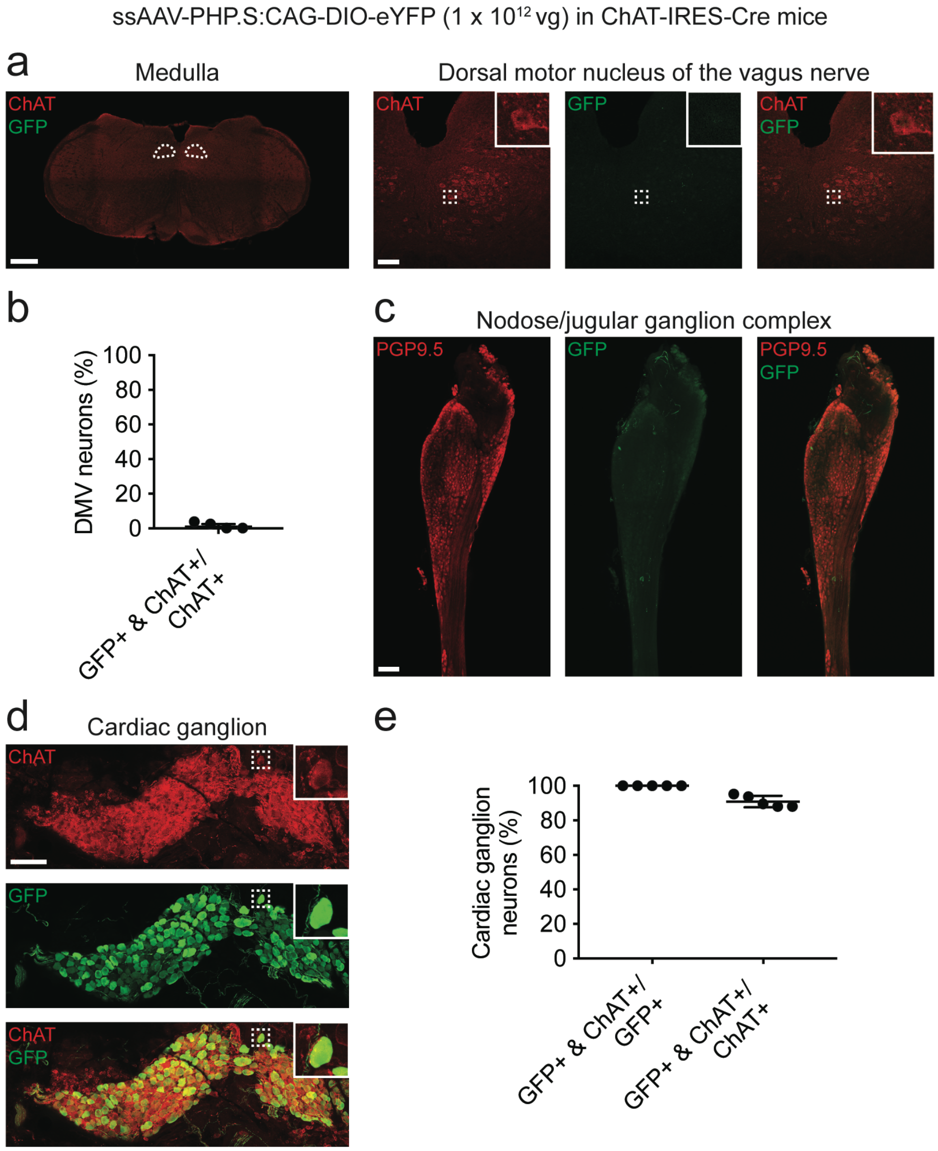


**Supplementary Figure 3. AAV-PHP.S preferentially transduces peripheral versus central cholinergic neurons.** ssAAV-PHP.S:CAG-DIO-eYFP was systemically administered to ChAT-IRES-Cre mice at 1 x $10^{12}$ vg per mouse. Three weeks later, eYFP fluorescence was assessed using an antibody for GFP. (**a**) Single-plane confocal images of the medulla (left) and dorsal motor nucleus of the vagus nerve (DMV) (right) whole-mount stained with ChAT (red) and GFP (green). White dashed ovals in the medulla show the location of the DMV. White dashed boxes in the DMV images indicate location of higher magnification images in white boxes. (**b**) Percentage of DMV neurons expressing GFP and ChAT over those expressing ChAT. (**c**) MIP images of the nodose/jugular ganglion complex whole-mount stained with PGP9.5 (red) and GFP (green). (**d**) MIP images of a cardiac ganglion from a heart whole-mount stained with PGP9.5 (red) and GFP (green). White dashed boxes indicate location of higher magnification images in white boxes. (**e**) Percentage of cardiac ganglion neurons expressing GFP and ChAT over those expressing GFP or ChAT, indicating specificity or efficiency of viral transduction, respectively. *n* = 4 mice (**b**) and

5 mice (**e**); mean ± s.e.m.. Scale bars are 500 μm (**a** (left)), 100 μm (**a** (right), **c**, **d** (right), and 1 mm (**d** (left)).

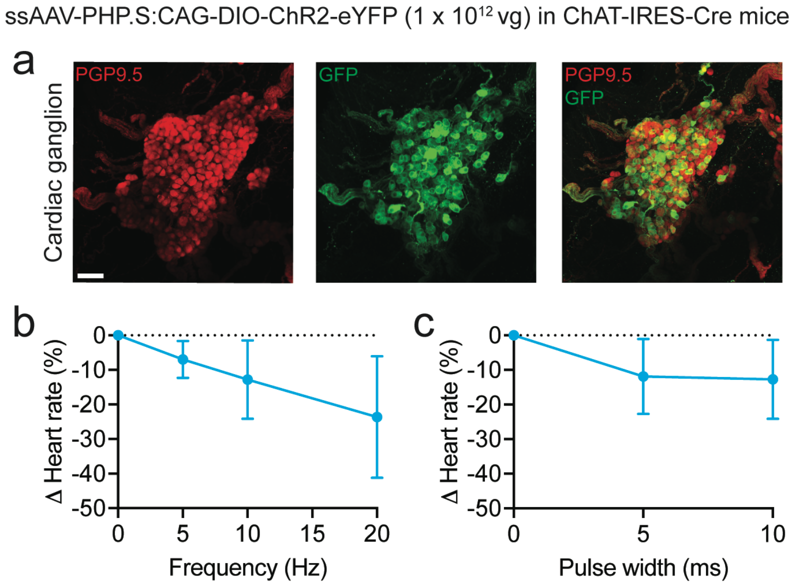


**Supplementary Figure 4. AAV-PHP.S for selective delivery of ChR2 to peripheral cholinergic neurons in the IPV-GP.** ssAAV-PHP.S:CAG-DIO-ChR2-eYFP was systemically administered to ChAT-IRES-Cre mice at 1 x $10^{12}$ vg per mouse. Experiments were performed 5 weeks post-injection. (**a**) MIP images of a cardiac ganglion from a heart whole-mount stained with PGP9.5 (red) and GFP (green). (**b**, **c**) Dose response curves summarizing the effects of altering stimulation frequency (**b**) and pulse width (**c**) on heart rate in Langendorff-perfused hearts. $n$ = 3 mice (**b**, **c**); mean ± s.e.m.. Scale bar is 100 μm (**a**).
