# Supplementary material for: Identification of peripheral neural circuits that regulate heart rate using optogenetic and viral vector strategies
Source: Nat Commun. 2019 Apr 26;10:1944. doi: 10.1038/s41467-019-09770-1 (PMC6486614; doi:10.1038/s41467-019-09770-1)
Supplement: Supplementary file 3 — Reporting Summary [file 41467_2019_9770_MOESM3_ESM.pdf]

nature research

Corresponding author(s): Kalyanam Shivkumar, Viviana Gradinaru

Reporting Summary

Nature Research wishes to improve the reproducibility of the work that we publish. This form provides structure for consistency and transparency in reporting. For further information on Nature Research policies, see Authors & Referees and the Editorial Policy Checklist.

Statistical parameters

When statistical analyses are reported, confirm that the following items are present in the relevant location (e.g. figure legend, table legend, main text, or Methods section).

n/a	Confirmed	
☐	☒	The exact sample size (*n*) for each experimental group/condition, given as a discrete number and unit of measurement
☐	☒	An indication of whether measurements were taken from distinct samples or whether the same sample was measured repeatedly
☐	☒	The statistical test(s) used AND whether they are one- or two-sided<br>*Only common tests should be described solely by name; describe more complex techniques in the Methods section.*
☒	☐	A description of all covariates tested
☐	☒	A description of any assumptions or corrections, such as tests of normality and adjustment for multiple comparisons
☐	☒	A full description of the statistics including central tendency (e.g. means) or other basic estimates (e.g. regression coefficient) AND variation (e.g. standard deviation) or associated estimates of uncertainty (e.g. confidence intervals)
☐	☒	For null hypothesis testing, the test statistic (e.g. *F*, *t*, *r*) with confidence intervals, effect sizes, degrees of freedom and *P* value noted<br>*Give P values as exact values whenever suitable.*
☒	☐	For Bayesian analysis, information on the choice of priors and Markov chain Monte Carlo settings
☒	☐	For hierarchical and complex designs, identification of the appropriate level for tests and full reporting of outcomes
☒	☐	Estimates of effect sizes (e.g. Cohen's *d*, Pearson's *r*), indicating how they were calculated
☐	☒	Clearly defined error bars<br>*State explicitly what error bars represent (e.g. SD, SE, CI)*

Our web collection on statistics for biologists may be useful.

Software and code

Policy information about availability of computer code

Data collection	Data were collected in this study using: a Zeiss LSM 880; a custom-built lightsheet microscope; and a AD Instruments PowerLab 8/35 system.
Data analysis	Data were analyzed in this study using: neuTube; custom Matlab scripts; National Institutes of Health ImageJ; AD Instruments LabChart v7.0.1; Microsoft Excel 16.21.1.; and GraphPad Prism 8.0.1.

For manuscripts utilizing custom algorithms or software that are central to the research but not yet described in published literature, software must be made available to editors/reviewers upon request. We strongly encourage code deposition in a community repository (e.g. GitHub). See the Nature Research guidelines for submitting code & software for further information.

Data

Policy information about availability of data

All manuscripts must include a data availability statement. This statement should provide the following information, where applicable:

- Accession codes, unique identifiers, or web links for publicly available datasets
- A list of figures that have associated raw data
- A description of any restrictions on data availability

All figures have associated raw data. The data that support the findings of this study are available from the corresponding authors upon reasonable request.

April 2018

Field-specific reporting

Please select the best fit for your research. If you are not sure, read the appropriate sections before making your selection.

☒ Life sciences ☐ Behavioural & social sciences ☐ Ecological, evolutionary & environmental sciences

For a reference copy of the document with all sections, see nature.com/authors/policies/ReportingSummary-flat.pdf

Life sciences study design

All studies must disclose on these points even when the disclosure is negative.

Sample size	No statistical methods were used to predetermine sample sizes. Samples sizes were chosen based on preliminary data that suggested a large effect size and based on published studies using similar techniques.
Data exclusions	Two animals in which optogenetic stimulation of the inferior pulmonary vein-ganglionated plexus was attempted were excluded from data analysis owing to failed Langendorff-perfusion of the heart.
Replication	Experimental findings were replicated in multiple animals for both imaging and functional studies.
Randomization	Randomization was not relevant for the functional studies as each animal served as its own control.
Blinding	Blinding was not performed for the functional studies during data collection or analysis as each animal served as its own control.

Reporting for specific materials, systems and methods

Materials & experimental systems

n/a	Involved in the study	
☐	☒	Unique biological materials
☐	☒	Antibodies
☒	☐	Eukaryotic cell lines
☒	☐	Palaeontology
☐	☒	Animals and other organisms
☒	☐	Human research participants

Methods

n/a	Involved in the study	
☒	☐	ChIP-seq
☒	☐	Flow cytometry
☒	☐	MRI-based neuroimaging

Unique biological materials

Policy information about availability of materials

Obtaining unique materials	Novel capsid sequences have been deposited to GenBank and viral plasmids have been deposited to Addgene.

Antibodies

Antibodies used	All antibodies used in the study are reported in the Methods section. Primary antibodies used were: rabbit anti-protein gene product 9.5 (Abcam, ab108986); rabbit anti-hyperpolarization-activated cyclic nucleodtide-gated potassium channel 4 (Alomone Labs, APC-052); chicken anti-green fluorescent protein (Aves, GFP-1020); sheep anti-tyrosine hydroxylase (Millipore Sigma, AB1542); goat anti-choline acetyltransferase (Millipore Sigma, AB144P); and rabbit anti-choline acetyltransferase (Millipore Sigma, AB143). Secondary antibodies used were: donkey anti-rabbit Cy3 (Jackson ImmunoResearch, 711-165-152); donkey anti-chicken 647 (Jackson ImmunoResearch, 703-605-155); donkey anti-sheep Cy3 (Jackson ImmunoResearch, 713-165-003); and donkey anti-goat Cy3 (Jackson ImmunoResearch, 705-165-003).
Validation	Validation of primary antibodies is listed on the manufacturers' websites and on CiteAb.

Animals and other organisms

Policy information about studies involving animals; ARRIVE guidelines recommended for reporting animal research

Laboratory animals	All laboratory mice used in this study are reported in the Methods section. ChAT-IRES-Cre, TH-IRES-Cre, Ai32, C57BL/6J, and Ai14 mice were used in this study. ChAT-ChR2-eYFP and TH-ChR2-eYFP mice were created by crossing Ai32 mice with ChAT-IRES-Cre

or TH-IRES-Cre mice, respectively. TH-tdTomato mice were created by crossing Ai14 mice with TH-IRES-Cre mice. Both male and female mice older than 6 weeks were used.

Wild animals	The study did not involve wild animals.
Field-collected samples	The study did not involve samples collected from the field.
