# Supplementary material for: Identification of peripheral neural circuits that regulate heart rate using optogenetic and viral vector strategies
Source: Nat Commun. 2019 Apr 26;10:1944. doi: 10.1038/s41467-019-09770-1 (PMC6486614; doi:10.1038/s41467-019-09770-1)
Supplement: Supplementary file 4 — Description of Additional Supplementary Files [file 41467_2019_9770_MOESM4_ESM.pdf]

Description of Additional Supplementary Files

File Name: Supplementary Movie 1
Description: Movie corresponds to Figure 1c. 3D confocal projection (1200 µm z-stack) of the dorsal side of a whole iDISCO-cleared heart with PGP9.5 staining (gray).

File Name: Supplementary Movie 2
Description: Movie corresponds to Figure 1. 3D projection (5590 µm z-stack) of a whole iDISCO-cleared heart with PGP9.5 staining (green). The heart was imaged with a lightsheet microscope.

File Name: Supplementary Movie 3
Description: Movie corresponds to Figure 1c. Confocal z-stack (785 µm zstack) through the left ventricle of a whole iDISCO-cleared heart with PGP9.5 staining (gray). A dense network of nerve fibers is observed from the epi- to endocardium.

File Name: Supplementary Movie 4
Description: Movie corresponds to Supplementary Figure 2b. 3D confocal projection (402 µm z-stack) of the left atrium of a PACT-cleared heart in which cholinergic nerve fibers (gray) were virally labelled.

File Name: Supplementary Movie 5
Description: Movie corresponds to Supplementary Figure 2c. 3D confocal projection (1000 µm z-stack) of the dorsal atrial wall of a PACT-cleared heart in which noradrenergic neurons (gray) in cardiac ganglia were endogenously labelled.

File Name: Supplementary Movie 6
Description: Movie corresponds to Supplementary Figure 2c. 3D confocal projection (85 µm z-stack) of the ventricle of a PACT-cleared heart in which noradrenergic nerve fibers (gray) were endogenously labelled.

File Name: Supplementary Movie 7
Description: Movie corresponds to Figure 5c. 3D confocal projection (173 µm z-stack) of cardiac-projecting neurons in the LSG labeled with CTB-488.
