# Supplementary material for: Identification of peripheral neural circuits that regulate heart rate using optogenetic and viral vector strategies
Source: Nat Commun. 2019 Apr 26;10:1944. doi: 10.1038/s41467-019-09770-1 (PMC6486614; doi:10.1038/s41467-019-09770-1)
Supplement: Supplementary file 2 — Peer Review [file 41467_2019_9770_MOESM2_ESM.pdf]

Reviewers' comments:

Reviewer #1 (Remarks to the Author):

This is an interesting manuscript that describes how to use optogenetic approaches to image the anatomy of the cardiac nervous system as well as how to use those approaches to dissect functional circuits within living heart tissue. There is a nice mix of technology development and mechanistic insights via an anatomic/functional analysis of the role of the cardiac IPV-GP. Very nice work is presented yet there are multiple areas where the manuscript can be improved, as explained below.

The clearing of mouse hearts using iDISCO is a new development and the results shown in Figure 1 are impressive. In Figure 1a it is interesting that PGP9.5 staining was done before clearing. Can the authors provide data regarding the loss of PGP9.5 immunofluorescence that results from iDISCO (ie, fluorescence signals for PFA-fixed then stained tissue compared to stained then iDISCO-cleared tissue). It is important to quantify how clearing approaches do or do not affect fluorescent probes.

It is interesting that secondary staining with GFP was required to amplify ChR2-eYFP fluorescence (ie, Figure 3a). Is this because iDISCO attenuated the fluorescence of ChR2-eYFP? Please consider recent work of Moreno, et al., available on bioRxiv (doi: https://doi.org/10.1101/451450) that reports high fluorescence of ChR2-eYFP (without GFP amplification) in a mouse model that is almost identical to that used by the authors.

In addition to the Yokoyama 2017 publication please also consider an additional publication that shows CLARITY clearing of a mouse heart (Figure 6i in Hsueh, Scientific Reports 2017). Figure 1 of Hsueh 2017 also reports the amount of protein loss after clearing. How does this compare to iDISCO?

Nearly-selective expression of ChR2 in peripheral neurons was accomplished using AAV-PHP.S to provide for specific stimulation of postganglionic cardiac neurons. Nearly-selective expression was confirmed via MIP confocal imaging (Supp Figure 2). Additional evidence for the selectivity of this viral approach would be to repeat the functional experiments (Supp Figure 2) after severing the cardiac vagal inputs, as was done for the experiments shown in Figure 4. Can data from such studies be provided?

A large portion of the manuscript is devoted to investigations of cholinergic neurons and the IPV-GP. The selectivity of the Cre-Lox system (transgenic model and the viral approach) for expression of ChR2 in cholinergic neurons is not assessed in a direct manner. The primary assessment is indirect, in that the authors report an overlay of PGP9.5 fluorescence and GFP-amplified ChR2-eYFP fluorescence. This is generally convincing yet quantitative assessment of co-location of ChR2-eYFP with ChAT in the heart would seal the deal. For example, the Moreno, et al., preprint shows co-localization of ChR2-eYFP with ChAT in cardiac neurons in a mouse model almost identical to that used by the authors. Indeed, the authors used ChAT immunohistochemistry for the dorsal motor nucleus in the medulla (Supp Figure 1a) yet there are no ChAT images shown for the cardiac ganglia. Such definitive proof of selective expression is particularly important for the ChAT-IRES-Cre transgenic mice transfected with AAV-PHP.S

Figure 3c: heart rate appears to be lower after cessation of illumination. Why? At 20Hz stimulation the R wave amplitude also drops after cessation of illumination. This is also true for 10Hz in Figure 3i. Is this typical? Please report the average response of the hearts after cessation of illumination. Is the post-illumination response dependent upon the duration of illumination or other illumination parameters?

Figure 6a: heart rate does not return to baseline levels. How long before it does?

For all experiments please report the illumination area. Irradiance values in mW/mm^2 will be more

informative than the currently reported power levels in mW. What might be the expected levels (increase in degC) of tissue warming if the power was directed onto a small area (ie, high irradiance)? Could slight warming above physiologic temp increase neuron firing rate and release of neurotransmitter?

How does the heart rate response to optical stimulation of the paravertebral ganglia compare to electrical stimulation of the ganglia? TH-ChR2-eYFP mice were used for the stellate experiments. It is possible that preganglionic neurons in the stellate were also stimulated? Such synapses are presumably nicotinic, yet is it safe to assume that there is no expression at all of ChR2 in those preganglionic neurons? Could ChAT + TH co-staining reveal the synaptic maps (re: Figure 5) and possibly indicate potential cross-over expression of ChR2?

Figures 3e, 3f, 4d, 4e, 4g, 6e, Supp 2b: none of these “dose-response” plots show a plateau or dose saturation. Can additional data be added to indicate maximal response levels?

Reviewer #2 (Remarks to the Author):

This is an interesting and technically impressive study using opticogenetic stimulation and genetic labeling to map autonomic innervation of the heart in mice. Inquiry into the anatomy and physiology of heart rate and conduction control by the autonomic nervous systems represents some of the oldest questions in ‘modern’ biology, and the current study adds some interesting new data. It also offers a new twist on these investigations using modern tools that, though technically challenging, may be used by others in the future. I have some questions, outlined below, on the fidelity of the systems, and whether the differences in optical studies and standard electrical stimulation studies provide true insights into the biology of the autonomic system, or may have less exciting, perhaps trivial, explanations due to imperfect expression or optical capture in the mouse models. Further comments follow:

why is HCN4 immunodetection adequate for defining the SAN/AVN? HCN4 is expressed in other cardiac tissues, including atrial myocardium. Furthermore, HCN4 expression may increase in response to injury, suggesting this approach could be misleading in the future studies foreshadowed in the Results section (e.g. MI and nerve sprouting) describing findings in Fig 1.

Were some neurons PGP9.5-? if so, why, given this is a pan-neuronal marker? if this marker has incomplete fidelity, what are the implications for your subsequent conclusions?

Last paragraph, page 5 - Could the lack of AVN conduction effect also mean that labeling and/or optical capture were incomplete? How can you rule out these possibilities?

Please show absolute heart rates in addition to percent change

Why is mixed recruitment of efferents/afferents by electrical stimulation more effective at rapidly increasing HR compared to optical pacing that is designed to only stimulate a pure population of nerves? Fig 4c - Given the relative delay in the optical verus electrical stimulation response, I wonder what is the evidence that the slower HR change with optical stimulation is not due to incomplete expression of the optically activated channel or a phased or delayed pattern of recruitment? How do your results unravel the previous findings re electrical stimulation of the PV ganglia (i.e. bradycardia followed by tachycardia), as mentioned in the Discussion?

The post vagotomy experiments with a different response to optical (none) and electrical (HR slowing) is fascinating. Nevertheless, i wonder how you can be sure it is not due to lack of adequate expression/capture/recruitment using the optical method? For example, an electrode recording showing an impulse with optical stimulation in the rostral limb of the severed vagus would strengthen your conclusion that the biology, rather than technical aspects of your approach, was driving your findings.

Paragraph 2, page 10 - The contrast between findings on sympathetic neurons in canines (post ganglionic neurons mostly in the middle cervical ganglia of the PV chain) versus mice (showing a craniomedial localization) might be due to species differences.
Minor
a minimum of 3 beats is excessively vague, arbitrary and, perhaps, insufficient to represent actual rates (i.e. 3 is a small number).

Reviewer #3 (Remarks to the Author):

In this well-written manuscript the authors present a novel tool for studying the role of parasympathetic and sympathetic nervous system in murine cardiac electrophysiology using optogenetics. They use parasympathetic and sympathetic specific Cre lines in combination with AAV driven fluorescent proteins to visualize the cardiac autonomic nervous system. Moreover, using tissue-specific ChR2 expression they specifically stimulate either the parasympathetic or sympathetic nervous system and investigated the effect on heart rate. The authors conclude to have provided novel insight into neural regulation of heart rate and tools for studying neuronal cardiac circuits or neural control of other organs.

comments

• The 3D visualizations of the neural circuit anatomy are beautiful but passive (movies showing pre-defined angles and magnifications), it would be great if the authors could provide files where the user can decide at what angle and magnification the 3D reconstructions are visualized. Is this possible? 3D pdf for example?

• Figure 2 and labeling of the cholinergic neurons. This part is confusing. Please explain the function of the human Synapsin I promotor. What is the reason for using the two component expression system? Please elaborate. Why not placing Cre directly under control of the Synapsin I promotor? Figure 2c shows results of an entirely different expression system. Please explain. How did the authors verify that labeling was indeed restricted to the cholinergic neurons? Low levels of Cre expression outside these neurons would generate aspecific labeling. Please provide additional data verifying specificity using an independent marker.

• The authors use multiple fluorescent proteins to visualize the parasympathetic nervous system. Please explain why.

• Figure 2D. It is unclear how the sections are oriented. I suggest to add an overview section indicating the location of the sections shown. Moreover, Hcn4 is used to mark the Sinus node and AV node. To me, however, the Hcn4 staining seems stronger outside the areas marked with the dashed white lines than inside. The AV node does not seem to be labeled. Please address this issue.

• Page 5. The authors use ChR2-enhanced yellow fluorescent protein but switch to GFP in their descriptions, which is confusing. “rostral” is archaic, cranial or anterior is more commonly used.

• Figure 4 C. The authors compare optical stimulation of the right vagal nerve with electrical stimulation of the right vagal nerve. The approaches differ in 2 ways. 1) Only a part of the RVNS is stimulated optically whereas the whole RVNS is stimulated during electrical stimulation, and 2) optical stimulation in itself differs from electrical stimulation. Why didn’t the authors compared electrical stimulation with optical stimulation of both the efferent and afferent fibers in the vagal nerve? This allows for discriminating the different responses to the different approaches.

• The authors report p wave fractionation during IPV-GP stimulation. Do the authors mean a biphasic p wave? It is known that the right and left atrium activation with a delay of about 5 ms. The activation wave propagates from the right atrium towards the left atrium via a dorsal myocardial connection. Do the authors think this delay between right and left atrium is increased during IPV-GP stimulation? Or does IPV-GP stimulation affect atrial conduction?

• Page 5 line 31. The authors mention that the nerve fiber passes through the AV node but does not synapse. Where is the nerve going? Was ventricular conduction affected?

• On page 7 Figure 4G is discussed before Figure 4F. Please adjust the figure order to the text.

• On page 8 and Figure 6, please provide validation of the specificity of the TH Cre driver.

• Discussion conclusion 3, it is not clear how the authors arrive at this conclusion. Please better explain. “central mechanisms”?

• References: In some cases the author list is abbreviated and in other case it is not. Please adjust to the correct style.

NCOMMS-18-29234A - Identification of peripheral neural circuits that regulate heart rate using optogenetic and viral vector strategies

Reviewers' comments:

We thank the editor and reviewers for their thoughtful review of our manuscript and for the opportunity to revise our work. We feel that in addressing the comments raised by the reviewers the manuscript has been significantly improved.

Reviewer #1 (Remarks to the Author):

This is an interesting manuscript that describes how to use optogenetic approaches to image the anatomy of the cardiac nervous system as well as how to use those approaches to dissect functional circuits within living heart tissue. There is a nice mix of technology development and mechanistic insights via an anatomic/functional analysis of the role of the cardiac IPV-GP. Very nice work is presented yet there are multiple areas where the manuscript can be improved, as explained below.

We thank the reviewer for careful reading of the manuscript and providing constructive comments. We have addressed the reviewer's comments point-by-point below.

The clearing of mouse hearts using iDISCO is a new development and the results shown in Figure 1 are impressive. In Figure 1a it is interesting that PGP9.5 staining was done before clearing. Can the authors provide data regarding the loss of PGP9.5 immunofluorescence that results from iDISCO (ie, fluorescence signals for PFA-fixed then stained tissue compared to stained then iDISCO-cleared tissue). It is important to quantify how clearing approaches do or do not affect fluorescent probes.

We performed additional experiments to compare hearts that were whole-mount stained (fixation followed by staining) and hearts that underwent the iDISCO protocol. The whole-mount stained hearts and iDISCO-cleared hearts were stained identically with the same concentrations of antibodies and for the same duration of time. In both whole-mount stained hearts and iDISCO cleared hearts, we observed dense innervation and similar patterns of PGP9.5 labeling on the epicardial surface (Supplemental Figure 1b and d). Importantly, following the iDISCO protocol, small nerve fiber bundles were preserved. The advantage of the iDISCO protocol versus whole-mount staining is the ability to label and visualize molecular and cellular structures in large tissue volumes[1]. In whole-mount stained hearts we were able to visualize fibers only within the first 100-200 µm of the epicardium (Supplemental Figure 1b), whereas in iDISCO-cleared hearts we were able to visualize fibers throughout the entire thickness of the myocardium from epi- to endocardium (Supplemental Figure 1d and Supplemental Movie 3).

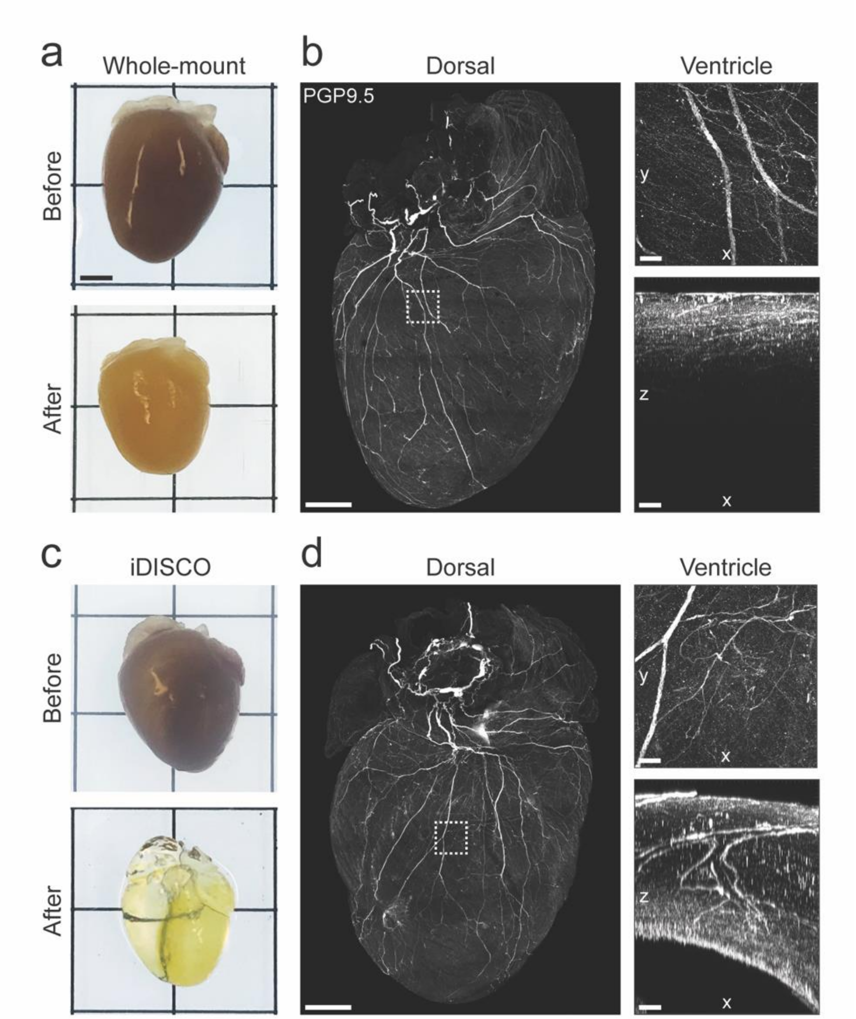

Supplemental Figure 1. Whole-mount stained heart versus iDISCO-cleared heart. (a) A heart before (top) and after (bottom) whole-mount staining. (**b**) 3D confocal projection of the dorsal side of a heart (2000 µm z-stack) whole-mount stained with PGP9.5 (gray). Insets show a MIP image of the left ventricular wall (top right) and a 1000 µm-thick 3D projection of the left ventricular wall (bottom right). (**c**) A whole heart (top) was rendered transparent (bottom) using the iDISCO protocol. (**d**) 3D confocal projection of the dorsal side of an iDISCO-cleared heart (2000 µm z-stack) stained with PGP9.5 (gray). Insets show a MIP image of the left ventricular wall (top right) and a 1000 µm-thick 3D projection of the left ventricular wall (bottom right). In contrast to whole-mount stained hearts, nerve fibers could be visualized throughout the entire thickness of myocardium in iDISCO-cleared hearts. Scale bars are 2 mm (**a**, **c**), 1 mm (**b** (left), **d** (left)), and 100 µm (**b** (right), **d** (right)).

Fluorescence intensity was lower in the iDISCO-cleared hearts as compared to the whole-mount stained hearts (Response Figure 1). However, we cannot comment on whether this is due to a loss of protein or the fact that antibodies penetrate much deeper (and, therefore, do not saturate antigen binding sites on the surface) in iDISCO-cleared tissues. Regardless, we do not see this as a major limitation of using iDISCO for characterizing innervation of the heart.

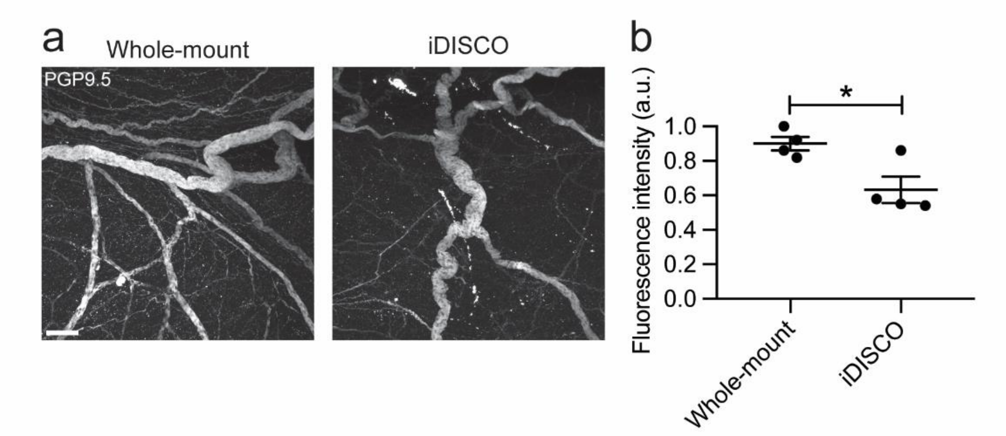

Response Figure 1. Fluorescence intensity in whole-mount stained hearts versus iDISCO-cleared hearts. (**a**) Representative MIP confocal images from the base of a whole-mount stained heart and an iDISCO-cleared heart with PGP9.5 staining (gray). (**b**) Mean PGP9.5 intensity from the base of whole-mount stained hearts versus iDISCO-cleared hearts (t_6 = 3.119; **P* = 0.0206). Note staining conditions were identical across all hearts and microscope laser settings remained constant across all images. *n* = 4 hearts (**b**). mean ± s.e.m.; unpaired, two-tailed *t*-test. Scale bar is 100 μm (**a**).

It is interesting that secondary staining with GFP was required to amplify ChR2-eYFP fluorescence (ie, Figure 3a). Is this because iDISCO attenuated the fluorescence of ChR2-eYFP? Please consider recent work of Moreno, et al., available on bioRxiv (doi: https://doi.org/10.1101/451450) that reports high fluorescence of ChR2-eYFP (without GFP amplification) in a mouse model that is almost identical to that used by the authors.

The iDISCO protocol was developed for immunostaining applications[1], in contrast to other tissue clearing techniques such as ScaleA2[2], 3DISCO[3], SeeDB[4], CLARITY[5,6] (Supplemental Figure 2), and CUBIC[7] that are better suited for imaging endogenous fluorescence. The dehydration processes and use of dibenzyl ether as an optical clearing agent in the iDISCO protocol are known to attenuate endogenous fluorescence[1].

In our manuscript, the hearts in Figure 1, Supplemental Figure 1, and Supplemental Movies 1-3 were immunostained with the pan-neuronal marker PGP9.5 and cleared with the iDISCO protocol as we were interested in characterizing innervation of entire hearts in 3D. However, the tissues in Figures 2-6 and Supplemental Figures 3 and 4 were whole-mount stained (as described in the Methods section under Immunohistochemistry) and not cleared using the iDISCO protocol as our goal was to confirm ChR2-eYFP expression in defined cell types (i.e. ChAT and TH neurons). We have clarified in the figure legends whether tissues were iDISCO-cleared or whole-mount stained. The tissues from ChAT-ChR2-eYFP and TH-ChR2-eYFP mice were stained with a widely-used antibody against GFP (Aves Labs, GFP-1020; 545 citations on CiteAb) to amplify the detection of the endogenous ChR2-eYFP protein and improve the signal-to-noise ratio. As shown in Response Figure 2, endogenous ChR2-eYFP signals were weak compared to those of GFP, even when using much higher laser power (100% laser power for imaging endogenous ChR2-eYFP versus 6% laser power for imaging GFP). Additionally, the use of high laser power results in more background autofluorescence.

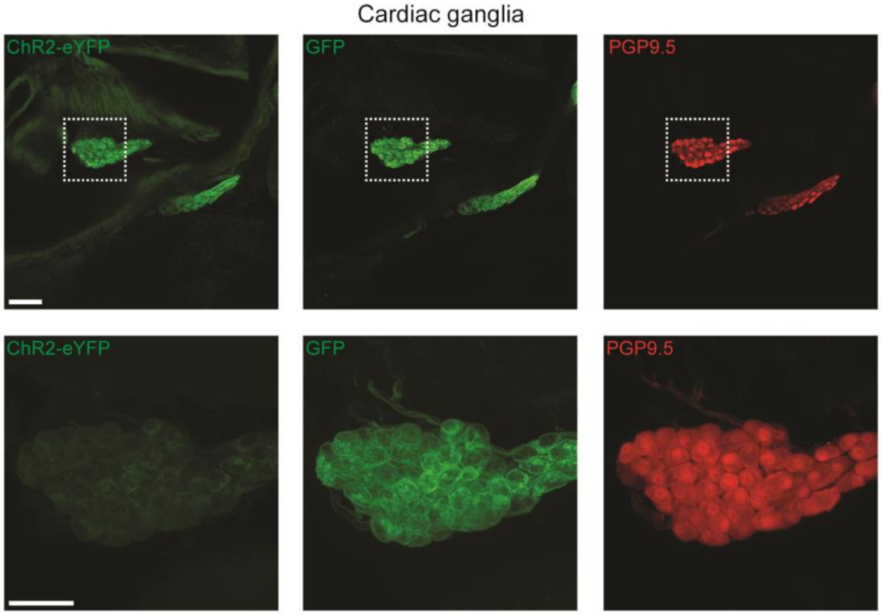

Response Figure 2. **ChR2-eYFP versus GFP signals in cardiac ganglia**. Confocal images showing native ChR2-eYFP signal versus GFP staining, along with PGP9.5 staining, from the cardiac ganglia of a ChAT-ChR2-eYFP mouse. Inset shows higher magnification image of a cardiac ganglion. Note ChR2-eYFP signal was imaged at 100% laser power and GFP was imaged at 6% laser power for all images. Background autofluorescence from myocardial tissue is observed in upper left ChR2-eYFP panel and weak signals are observed in lower left ChR2-eYFP panel likely due to photobleaching. Scale bars are 100 µm (upper panels) and 50 µm (lower panels).

We thank the reviewer for bring to our attention the manuscript by Moreno and colleagues. In the manuscript by Moreno *et al.*, endogenous ChR2-eYFP fluorescence was imaged from a small volume of the atria. However, in our manuscript, we were concerned about background autofluorescence and photobleaching resulting from use of high laser power to image endogenous ChR2-eYFP signals over large tissues volumes such as entire hearts; thus, tissues were stained with an antibody against GFP.

In addition to the Yokoyama 2017 publication please also consider an additional publication that shows CLARITY clearing of a mouse heart (Figure 6i in Hsueh, Scientific Reports 2017). Figure 1 of Hsueh 2017 also reports the amount of protein loss after clearing. How does this compare to iDISCO?

Although protein loss with iDISCO has not been previously reported[1,8,9], Reiner *et al.* compared FoxP2 expression in the inferior olive of an iDISCO-cleared adult mouse brain with tissue sections from the same level in the original iDISCO manuscript[1]. The Fox2P expression pattern and the number of Fox2P-positive cells was consistent between the two methods and other publications. In addition, our data show similar patterns of PGP9.5 expression between whole-mount stained hearts and iDISCO-cleared hearts, including preservation of smaller fiber bundles (Supplemental Figure 1). Taken together, this suggests that protein loss is not a major limitation of using the iDISCO protocol for characterizing innervation of the heart.

Nearly-selective expression of ChR2 in peripheral neurons was accomplished using AAV-PHP.S to provide for specific stimulation of postganglionic cardiac neurons. Nearly-selective expression was confirmed via MIP confocal imaging (Supp Figure 2). Additional evidence for the selectivity of this viral approach would be to repeat the functional experiments (Supp Figure 2) after severing the cardiac vagal inputs, as was done for the experiments shown in Figure 4. Can data from such studies be provided?

In the mouse heart, cardiac ganglia are located on the dorsal surface around the pulmonary veins (Figures 1c and 3a)[10-12]. It is not possible to expose them surgically *in vivo*. Therefore, the experiments in Figure 3 and Supplemental Figure 4 (old Supplemental Figure 2) in which the inferior pulmonary vein-ganglionated plexus (IPV-GP) was optogenetically stimulated were performed in *ex vivo* Langendorff-perfused hearts (as indicated in the figure legends and described in the Methods section under Optogenetic stimulation and physiological measurements), whereas the experiments in Figure 4 in which the cervical vagus nerve was optogenetically stimulated were performed *in vivo.* In Figure 3 and Supplemental Figure 4 (old Supplemental Figure 2), when excising the heart from the thorax for the Langendorff preparation, vagal preganglionic fibers originating from the medulla in the brainstem were indeed transected. However, even after transection, the caudal end of the vagus nerve, which contains preganglionic fibers, can still be stimulated *in vivo* (Figure 4e) and in Langendorff-perfused hearts[13,14].

In addition to viral transduction efficiency, another potential reason for the smaller bradycardic response observed with optogenetic stimulation of the IPV-GP in Supplemental Figure 4 (old Supplemental Figure 2) compared to that in Figure 3 is that in ChAT-IRES-Cre mice injected with AAV-PHP.S:CAG-DIO-ChR2-eYFP only postganglionic cholinergic neurons were transduced (Supplemental Figure 3 (old Supplemental Figure 1)) and optogenetically stimulated, whereas in ChAT-ChR2-eYFP mice both vagal preganglionic fibers and postganglionic cholinergic neurons expressed ChR2-eYFP and were stimulated. Future studies will focus on leveraging engineered AAVs such as AAV-PHP.S[15-17] to selectively transduce defined cell types in the peripheral nervous system.

A large portion of the manuscript is devoted to investigations of cholinergic neurons and the IPV-GP. The selectivity of the Cre-Lox system (transgenic model and the viral approach) for expression of ChR2 in cholinergic neurons is not assessed in a direct manner. The primary assessment is indirect, in that the authors report an overlay of PGP9.5 fluorescence and GFP-amplified ChR2-eYFP fluorescence. This is generally convincing yet quantitative assessment of co-location of ChR2-eYFP with ChAT in the heart would seal the deal. For example, the Moreno, et al., preprint shows co-localization of ChR2-eYFP with ChAT in cardiac neurons in a mouse model almost identical to that used by the authors. Indeed, the authors used ChAT immunohistochemistry for the dorsal motor nucleus in the medulla (Supp Figure 1a) yet there are no ChAT images shown for the cardiac ganglia. Such definitive proof of selective expression is particularly important for the ChAT-IRES-Cre transgenic mice transfected with AAV-PHP.S

Most, if not all, commercially available antibodies against ChAT recognize a central rather than peripheral isoform of ChAT, labeling peripheral cholinergic neurons weakly[18]. An antibody has been developed against the peripheral isoform of ChAT[19]; however, it is not yet commercially available. Therefore, we used an antibody against ChAT in the medulla in the central nervous system and an antibody against PGP9.5 as a surrogate for ChAT in cardiac ganglia in the peripheral nervous system due to the weak labeling and high background associated with ChAT staining in the periphery.

We performed additional immunohistochemistry to address the reviewer's comment. Please see Response Figure 3 and Supplemental Figure 3 (old Supplemental Figure 1). In ChAT-IRES-Cre mice injected with ssAAV-PHP.S:CAG-DIO-eYFP, we observed strong co-localization of endogenous ChR2-eYFP with GFP and ChAT staining in cardiac ganglion neurons (Response Figure 3). The virus was highly efficient at transducing peripheral neurons in cardiac ganglia (91.0 ± 1.5) versus central neurons in the dorsal motor nucleus of the vagus (1.5 ± 0.9%) (Supplemental Figure 3b and e). Expression was also highly specific for cholinergic neurons in cardiac ganglia (100 ± 0% of GFP+ neurons were ChAT+) (Supplemental Figure 3d and e).

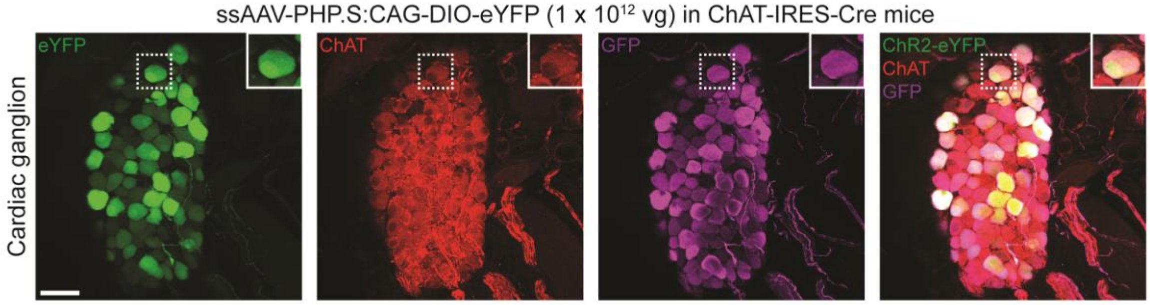

Response Figure 3. ssAAV-PHP.S:CAG-DIO-eYFP was systemically administered to ChAT-IRES-Cre mice at 1 x 10^{12} vg per mouse. Three weeks later, eYFP fluorescence was assessed. Representative single-plane confocal images of a cardiac ganglion with native eYFP signal (green) and ChAT (red) and GFP (magenta) staining. Scale bar is 50 µm.

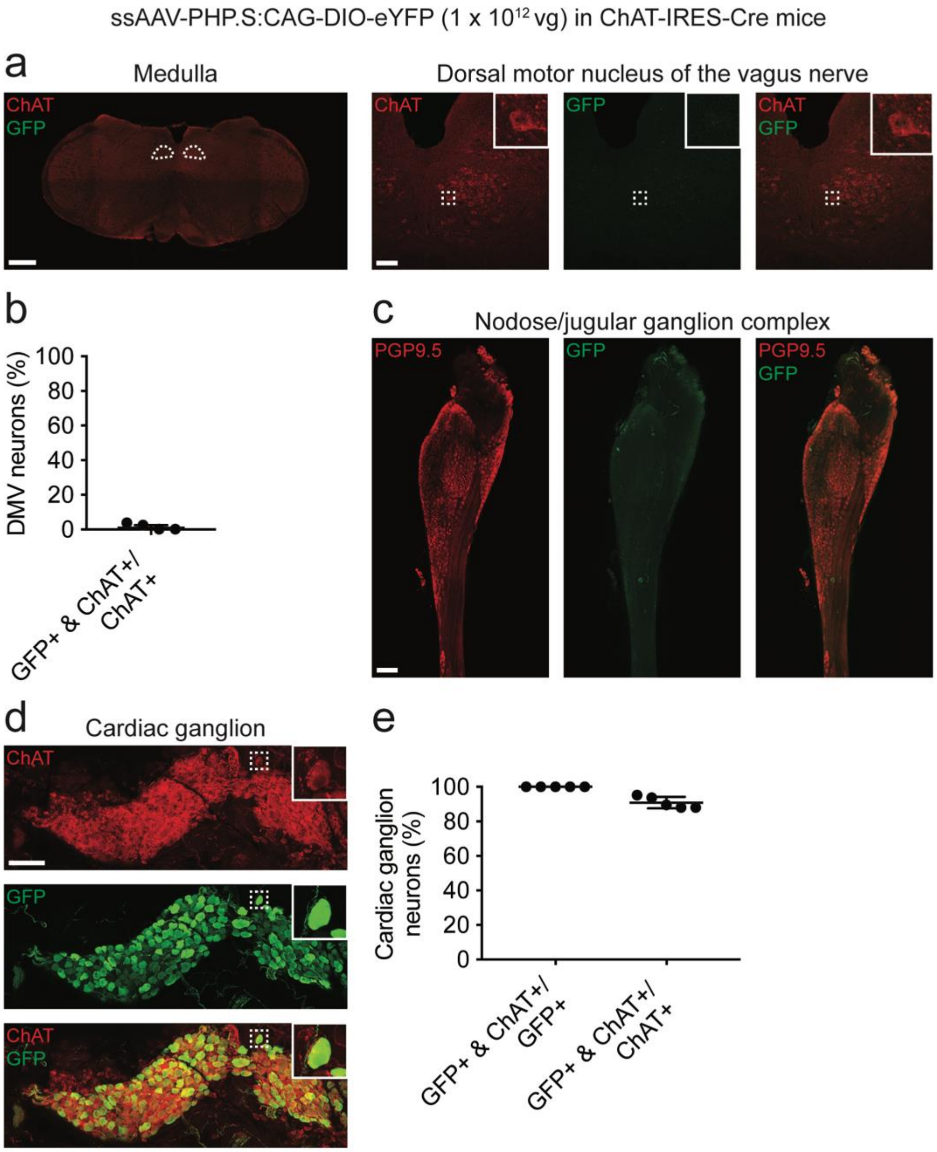

Supplemental Figure 3. AAV-PHP.S preferentially transduces peripheral versus central cholinergic neurons. ssAAV-PHP.S:CAG-DIO-eYFP was systemically administered to ChAT-IRES-Cre mice at 1 x 10^{12} vg per mouse. Three weeks later, eYFP fluorescence was assessed using an antibody for GFP. (**a**) Single-plane confocal images of the medulla (left) and dorsal motor nucleus of the vagus nerve (DMV) (right) whole-mount stained with ChAT (red) and GFP (green). White dashed ovals in the medulla show the location of the DMV. White dashed boxes in the DMV images indicate location of higher magnification images in white boxes. (**b**) Percentage of DMV neurons expressing GFP and ChAT over those expressing ChAT. (**c**) MIP images of the nodose/jugular ganglion complex whole-mount stained with PGP9.5 (red) and GFP (green). (**d**) MIP images of a cardiac ganglion from a heart whole-mount stained with PGP9.5 (red) and GFP (green). White dashed boxes indicate location of higher magnification images in white boxes. (**e**) Percentage of cardiac ganglion neurons expressing GFP and ChAT over those expressing GFP or ChAT, indicating specificity or efficiency of viral transduction, respectively. *n* = 4 mice (**b**) and

5 mice (**e**); mean ± s.e.m.. Scale bars are 500 µm (**a** (left)), 100 µm (**a** (right), **c**, **d** (right), and 1 mm (**d** (left)).

Figure 3c: heart rate appears to be lower after cessation of illumination. Why? At 20Hz stimulation the R wave amplitude also drops after cessation of illumination. This is also true for 10Hz in Figure 3i. Is this typical? Please report the average response of the hearts after cessation of illumination. Is the post-illumination response dependent upon the duration of illumination or other illumination parameters?

Following optogenetic stimulation of the IPV-GP, heart rate returned to baseline within 16.6 ± 4.4 s at 10 Hz and within 13.1 ± 2.7 s at 20 Hz (Response Figure 4c). These recovery times are in line with those reported by Moreno *et al.* (5 - 10 s; doi: https://doi.org/10.1101/451450). The heart rate recovery was not dependent on frequency or pulse width of stimulation. The duration of stimulation was 10 s and was not varied during the experiments. The absolute and relative change in heart rate at baseline, during stimulation, and following stimulation are shown in Response Figure 4a, b, d and e.

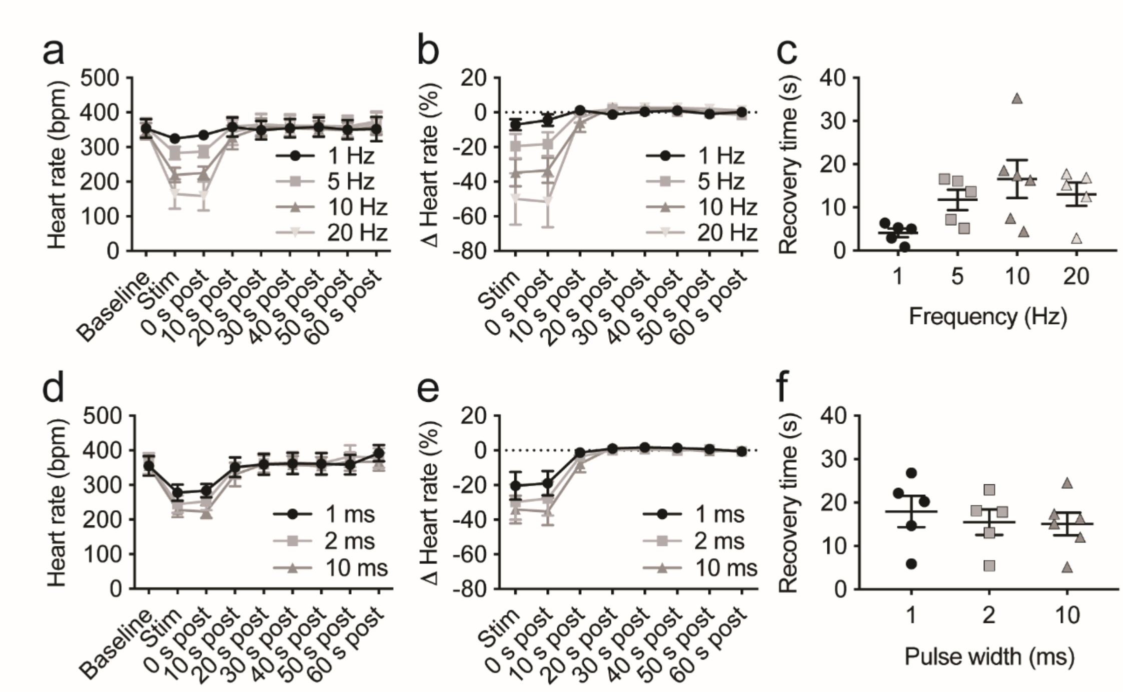

Response Figure 4. **Heart rate recovery following optogenetic stimulation of cholinergic neurons in the IPV-GP.** (**a**, **b**) Summary of the effects of altering stimulation frequency on the absolute (**a**) and delta heart rate response (**b**) before, during, and following optogenetic stimulation of the IPV-GP at 10 ms and 221 mW. (**c**) Summary of the time to recovery to baseline heart rate from **a** and **b** (F (1.470, 5.881) = 4.503, P = 0.0716). (**d**, **e**) Summary of the effects of altering stimulation pulse width on the absolute (**d**) and delta heart rate response (**e**) before, during, and following stimulation at 10 Hz and 221 mW. (**f**) Summary of the time to recovery to baseline heart rate from **d** and **e** (F (1.245, 4.981) = 0.3002, P = 0.6557). n = 6 mice (**a**-**f**). mean ± s.e.m.; mixed-effects ANOVA.

We used two electrodes in the bath to record a pseudo-electrocardiogram (ECG) and a quadripolar electrophysiology (EP) catheter in the left atrium and ventricle to record intracardiac electrograms. The change in R wave amplitude following stimulation is likely due to cardiac

motion—movement of the heart between the ECG electrodes and movement of the EP catheter within the heart. The change in R wave amplitude had no effect on any of our measurements.

Figure 6a: heart rate does not return to baseline levels. How long before it does?

Following optogenetic stimulation of the right stellate ganglion, heart rate returned to baseline within 18.9 ± 7.6 s at 10 Hz and 31.9 ± 13.7 s at 20 Hz (Response Figure 5c). Heart rate did not return to baseline in 2/7 mice at 10 Hz and 1/7 mice at 20 Hz. However, we waited for heart rate to stabilize before continuing the experimental protocol. The absolute and relative change in heart rate at baseline, during stimulation, and following stimulation are shown in Response Figure 5a, b, d and e.

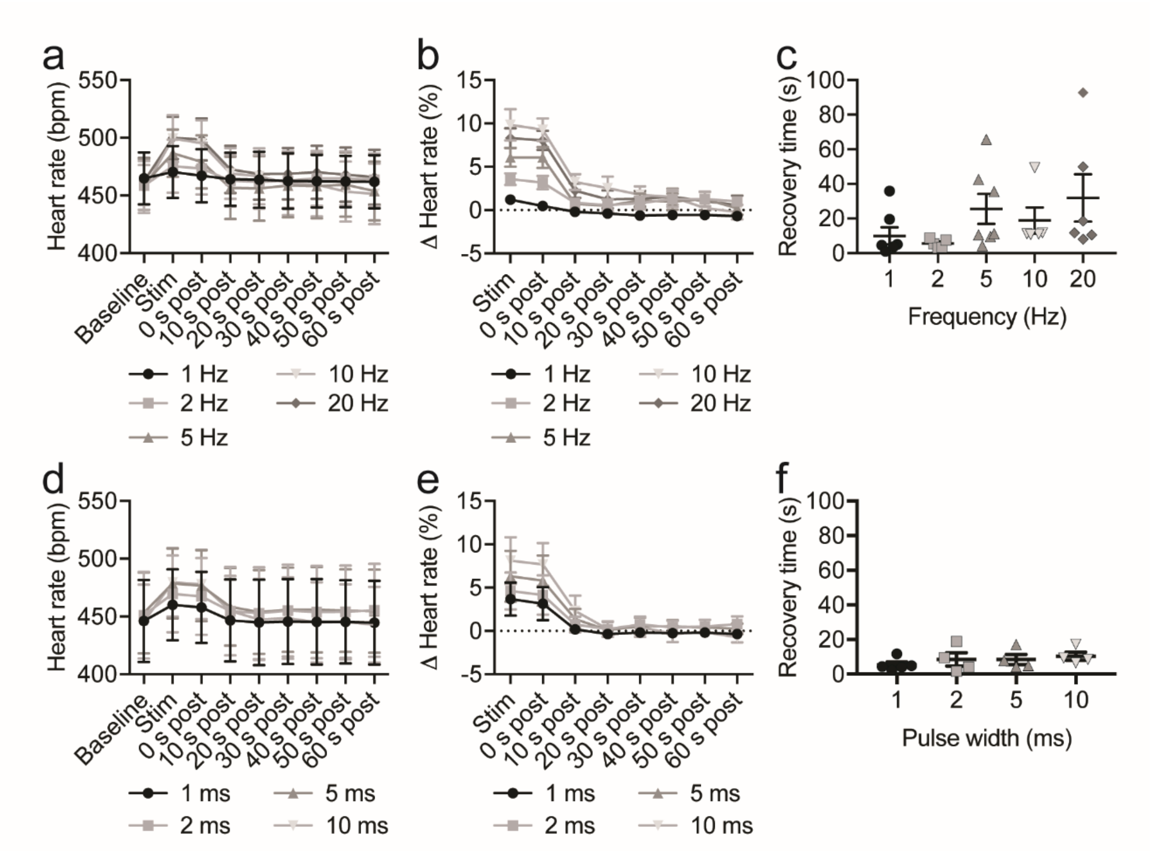

Response Figure 5. **Heart rate recovery following optogenetic stimulation of noradrenergic neurons in the right stellate ganglion (RSG).** (**a**, **b**) Summary of the effects of altering stimulation frequency on the absolute (**a**) and delta heart rate response (**b**) before, during, and following optogenetic stimulation of the RSG at 10 ms and 126 mW. (**c**) Summary of the time to recovery to baseline heart rate from **a** and **b** (F (1.673, 7.945) = 2.607, P = 0.1386). (**d**, **e**) Summary of the effects of altering stimulation pulse width on the absolute (**d**) and delta heart rate response (**e**) before, during, and following stimulation at 10 Hz and 126 mW. (**f**) Summary of the time to recovery to baseline heart rate from **d** and **e** (F (1.029, 3.088) = 3.591, P = 0.1521). n = 7 mice (**a**-**c**) and 5 mice (**d**-**f**). mean ± s.e.m.; mixed-effects ANOVA.

For all experiments please report the illumination area. Irradiance values in mW/mm^2 will be more informative than the currently reported power levels in mW. What might be the expected levels (increase in degC) of tissue warming if the power was directed onto a small area (ie, high irradiance)? Could slight warming above physiologic temp increase neuron firing rate and release of neurotransmitter?

Laser power was reported rather than irradiance because it is difficult to accurately measure the area of tissue illuminated. For all experiments, the optical fiber was positioned directly above (<1-2 millimeters) the tissue of interest. Laser power at the tip of the optical fiber was measured with an optical power meter.

In our Langendorff experiments, the temperature increase at the tip of the laser-coupled optical fiber at 221 mW, 20 Hz, and 10 ms was 1.1 ± 0.0 °C, which is well below the 6-10 °C increase in temperature that has been reported to be necessary for peripheral nerve stimulation[20]. We performed additional experiments to determine whether this increase in temperature could result in neuronal firing. In Langendorff-perfused wild-type mouse hearts in which there was no expression of ChR2, an optical fiber was positioned for focal illumination of the IPV-GP. The IPV-GP was stimulated at 221 mW, 20 Hz, and 10 ms. There was no change in heart rate with stimulation (Response Figure 6a and b). Using the same stimulation parameters in ChAT-ChR2-eYFP mice, heart rate decreased by 50.0 ± 14.9%. These data indicate that the slight increase in temperature was not responsible for neuronal firing in our preparation.

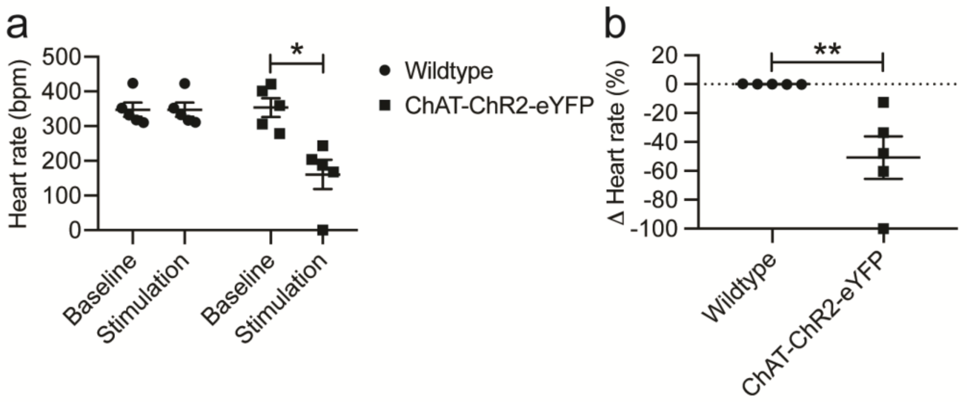

Response Figure 6. Heart rate response to optogenetic stimulation of the IPV-GP in wild-type and ChAT-ChR2-eYFP mouse hearts. A laser-coupled optical fiber was positioned for focal illumination of the IPV-GP in Langendorff-perfused wild-type versus ChAT-ChR2-eYFP mouse hearts. (**a**, **b**) Summary of the absolute (**a**) and delta heart rate response (**b**) to optogenetic stimulation of wild-type versus ChAT-ChR2-eYFP mouse hearts at 20 Hz, 10 ms, and 221 mW (t_4 = 0.5979, *P* = 0.5821 for wild-type baseline versus stimulation; t_4 = 3.033, **P* = 0.0387 for ChAT-ChR2-eYFP baseline versus stimulation; t_8 = 3.470, ***P* = 0.0084 for wild-type versus ChAT-ChR2-eYFP). *n* = 5 mice (**a**, **b**). mean ± s.e.m.; paired, two-tailed *t*-test (**a**) and unpaired, two-tailed *t*-test (**b**).

How does the heart rate response to optical stimulation of the paravertebral ganglia compare to electrical stimulation of the ganglia?

We performed additional experiments to compare electrical stimulation of the RSG in wild-type mice to optical stimulation in TH-ChR2-eYFP mice (Response Figure 7). In TH-ChR2-eYFP mice,

a laser-coupled optical fiber was positioned above the RSG for focal optical stimulation (as described in the Methods under Optogenetic stimulation and physiological measurements). In wild-type mice, a concentric bipolar electrode (FHC, 30324) coupled to a constant current stimulator (Grass, PSIU6 and Model S88) was positioned above the RSG for electrical stimulation. The current for electrical stimulation in wild-type mice was titrated to achieve an increase in heart rate at 10 Hz and 10 ms equivalent to that of optical stimulation at 126 mW, 10 Hz, and 10 ms in TH-ChR2-eYFP mice, which was defined as threshold current. The threshold current was 72 ± 9 µA. The frequency and pulse width response curves for optical versus electrical stimulation of the RSG were not significantly different.

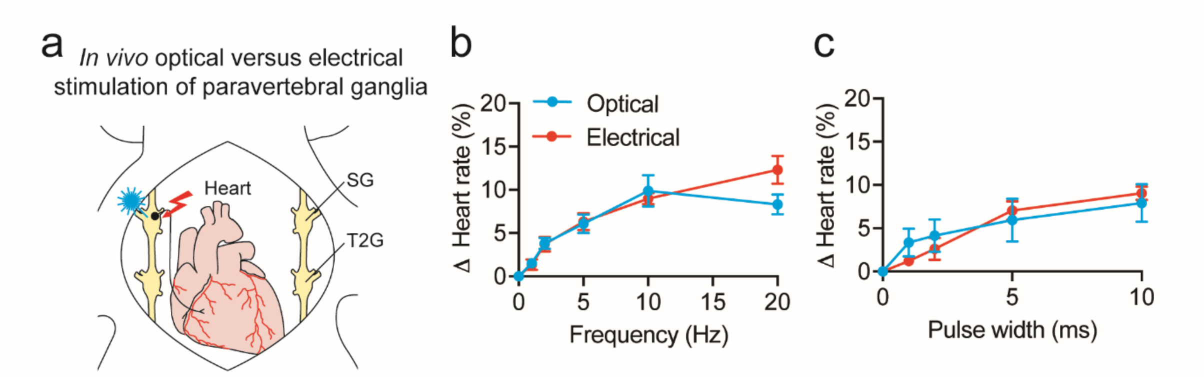

Response Figure 7. *In vivo* optogenetic versus electrical stimulation of the RSG. (**a**) Cartoon depicting optogenetic and electrical RSG stimulation strategy in TH-ChR2-eYFP and wild-type mice, respectively. The right paravertebral chain was surgically exposed in anesthetized mice and either light or electricity was used for RSG stimulation. (**b**) Frequency response curves summarizing the effects of optogenetic versus electrical RSG stimulation at 10 ms (t_{11} = 0.2395, P = 0.8151 at 1 Hz; t_{11} = 0.06439, P = 0.9498 at 2 Hz; t_{11} = 0.1467, P = 0.8860 at 5 Hz; t_{11} = 0.4398, P = 0.6686 at 10 Hz; and t_{11} = 2.050, P = 0.0650 at 20 Hz for optical versus electrical). (**c**) Pulse width response curves summarizing the effects of optogenetic versus electrical RSG stimulation at 10 Hz (t_8 = 1.047, P = 0.3255 at 1 ms; t_9 = 0.6464, P = 0.5342 at 2 ms; t_9 = 0.3787, P = 0.7137 at 5 ms; and t_9 = 0.4506, P = 0.6629 at 10 ms for optical versus electrical). n = 7 mice (**b** optical), 6 mice (**b** electrical, **c** optical), and 5 mice (**c** electrical). mean ± s.e.m.; paired, two-tailed t-test. T2G, second thoracic ganglion.

TH-ChR2-eYFP mice were used for the stellate experiments. It is possible that preganglionic neurons in the stellate were also stimulated? Such synapses are presumably nicotinic, yet is it safe to assume that there is no expression at all of ChR2 in those preganglionic neurons? Could ChAT + TH co-staining reveal the synaptic maps (re: Figure 5) and possibly indicate potential cross-over expression of ChR2?

While it is possible that there was non-specific expression of ChR2-eYFP in TH-ChR2-eYFP mice, our functional data suggest that sympathetic preganglionic fibers within the paravertebral chain did not express ChR2-eYFP. As the reviewer indicates, sympathetic preganglionic neurons are cholinergic[21] and ChR2-eYFP expression in TH-ChR2-eYFP mice should be restricted to neurons that express TH. Sympathetic preganglionic neurons that project to sympathetic postganglionic neurons innervating the heart are located in the T1-T6 segments of the spinal cord[22]. Axons from these sympathetic preganglionic neurons exit the ventral rami of the spinal cord and course up and down the paravertebral chain to synapse predominately on neurons in the craniomedial stellate ganglia (Figure 5c and d), which then project to the heart. Previous studies in large

animals have shown that electrical stimulation of the T1-T2 region of the paravertebral chain, which contains both sympathetic pre- and postganglionic fibers, elicits an increase in heart rate[23,24]. In our studies, optogenetic stimulation of the right stellate ganglion resulted in an increase in heart rate, whereas there was no response to stimulation of the right T2 ganglion and sympathetic preganglionic fibers that course through that region (Figure 6g). Therefore, we believe that the tachycardic response from right stellate ganglion stimulation was due to activation of postganglionic neurons projecting to the heart not preganglionic fibers.

Figures 3e, 3f, 4d, 4e, 4g, 6e, Supp 2b: none of these “dose-response” plots show a plateau or dose saturation. Can additional data be added to indicate maximal response levels?

The stimulation parameters for all the experiments were chosen based on previous studies of electrical[25-28] and optogenetic stimulation[29-31] of the cardiac autonomic nervous system.

Reviewer #2 (Remarks to the Author):

This is an interesting and technically impressive study using opticogenetic stimulation and genetic labeling to map autonomic innervation of the heart in mice. Inquiry into the anatomy and physiology of heart rate and conduction control by the autonomic nervous systems represents some of the oldest questions in ‘modern’ biology, and the current study adds some interesting new data. It also offers a new twist on these investigations using modern tools that, though technically challenging, may be used by others in the future. I have some questions, outlined below, on the fidelity of the systems, and whether the differences in optical studies and standard electrical stimulation studies provide true insights into the biology of the autonomic system, or may have less exciting, perhaps trivial, explanations due to imperfect expression or optical capture in the mouse models.

We thank the reviewer for thorough reading of the manuscript and insightful comments. We have addressed the reviewer’s comments point-by-point below.

Further comments follow:

Why is HCN4 immunodetection adequate for defining the SAN/AVN? HCN4 is expressed in other cardiac tissues, including atrial myocardium. Furthermore, HCN4 expression may increase in response to injury, suggesting this approach could be misleading in the future studies foreshadowed in the Results section (e.g. MI and nerve sprouting) describing findings in Fig 1.

The SA node and AV node were identified by a combination of anatomical landmarks (i.e., sulcus terminalis and superior vena cava for the SA node; base of the right atrium and interatrial septum for AV node) and HCN4 immunodetection.

HCN channels are expressed in cardiac and neural tissues and conduct the I_f current in heart and I_h current in neural tissues. As the reviewer correctly states, HCN4 is expressed in various cardiac tissues. However, while expression of the HCN1, HCN2 and HCN4 isoforms has been described in pacemaker cells, studies across many vertebrates indicate that HCN4 is the most highly expressed isoform in the SA node. For example, in the rabbit SA node, HCN4 comprises >81% of the HCN isoforms[32]. Genetic mutations in HCN4 have been shown to cause sinus node dysfunction in humans[33-36]. An expression study that evaluated the distribution of HCN transcript in the mouse heart indicates that 59% of the HCN transcript in the SAN is HCN4, while only 6% and 11% of HCN transcripts in the LA and RA, respectively, are HCN4[37]. Functionally, HCN4 is

also required for the generation of mature pacemaker potentials in the developing SA node during embryogenesis in mice and contributes to the majority of the SAN I_f at around 70-80%[38-40]. HCN1 is also expressed in the cardiac conduction system and does contribute to a component of I_f in the SAN[41], but is expressed in 73% of the LA and 58% of the RA in the mouse heart[37], making it less specific for the SA node. HCN2 is the dominant HCN transcript in ventricular myocardium.

Regarding the AV node, HCN4 has been shown to be expressed in the rabbit[42], rat[43] and human[44]. In humans, HCN4 is the most highly expressed HCN transcript in the AV conduction axis but below detection threshold in working myocardium[44]. The expression pattern of the HCN channels in the AVN has been shown to be similar to that of the SAN in mouse[37]. In addition, HCN4 is the target in mouse models for conditional knockout of a gene specific for the cardiac conduction system[39]. Based on the literature above, HCN4 appears to be the most highly expressed HCN isoform and specific marker of the cardiac conduction system including the SA and AV nodes across vertebrates. For these reasons, we utilized HCN4 immunodetection, in addition to well-known anatomical landmarks, to identify the SA and AV nodes in the mouse heart.

While increased channel expression and activity of HCN channels (particularly the HCN2 isoform) have been demonstrated in response to inflammation and neural injury in the peripheral nervous system, this has not been shown to be the case with myocardial injury or HCN4 channels, to our knowledge[45-52]. In future studies, upregulation of HCN channel expression could be assessed in neural tissues by co-localization of our pan-neuronal marker with HCN as well as in cardiac tissue by counterstaining with a myocyte marker.

Were some neurons PGP9.5-? if so, why, given this is a pan-neuronal marker? If this marker has incomplete fidelity, what are the implications for your subsequent conclusions?

PGP9.5 is a cytoplasmic neuron-specific protein[53] and is widely used as a pan-neuronal marker including in studies of cardiac innervation[54-59]. In our studies, we did not observe any neurons in cardiac ganglia or nodose ganglia that were PGP9.5-.

Last paragraph, page 5 - Could the lack of AVN conduction effect also mean that labeling and/or optical capture were incomplete? How can you rule out these possibilities?

While we cannot rule out that the lack of effect on AV conduction with optogenetic stimulation of the IPV-GP was due to incomplete expression of ChR2 or optical capture, our data suggest that this is not case. Almost all PGP9.5+ neurons (96.4 $\pm$ 1.2%) in the cardiac ganglia of ChAT-ChR2-eYFP mice were GFP+ (Figure 3b). The IPV-GP was also identified under a fluorescent stereomicroscope and a laser-coupled optical fiber was positioned above the ganglion for focal illumination. Further, since we were directly stimulating the cell bodies in the ganglion, fibers originating from this ganglion and potentially projecting to the AV node should have also been activated. Nonetheless, we have added a Limitation section in which this is mentioned.

Please show absolute heart rates in addition to percent change.

Absolute heart rate, in addition to the relative changes in heart rate, has now been added for all experiments in Supplemental Tables 1-3.

**Power (mW)** (*n* = 6)	**Baseline heart rate (bpm)**	**Stimulation heart rate (bpm)**	**Δ Heart rate (%)**
0	355.8 ± 37.7	355.1 ± 38.0	-0.2 ± 0.2
38	365.8 ± 34.1	307.2 ± 46.1	-16.0 ± 8.7
115	353.0 ± 35.4	228.3 ± 23.7	-31.8 ± 11.0
226	379.2 ± 35.6	200.3 ± 30.0	-45.9 ± 9.7
**Frequency (Hz)** (*n* = 6)			
1	353.5 ± 27.2	324.6 ± 13.2	-7.2 ± 3.3
5	357.8 ± 25.7	282.4 ± 18.3	-19.7 ± 7.1
10	347.9 ± 23.0	219.7 ± 21.0	-35.2 ± 8.0
20	351.2 ± 29.0	164.5 ± 42.9	-50.0 ± 14.9
**Pulse width (ms)** (*n* = 6)			
1	355.6 ± 28.5	278.1 ± 23.8	-20.4 ± 8.0
2	362.1 ± 30.4	244.8 ± 25.4	-29.7 ± 10.3
10	356.6 ± 25.5	227.1 ± 21.6	-34.4 ± 8.2
**Atropine** (*n* = 5)			
Pre	354.9 ± 35.3	224.2 ± 25.0	-33.5 ± 11.0*
Post	333.8 ± 37.7	333.0 ± 37.9	-0.3 ± 0.2

Supplemental Table 1. *Ex vivo* optogenetic stimulation of cholinergic neurons in the IPV-GP. Table corresponds to Figure 3. Dose response curves summarizing the effects of altering light pulse power, frequency, and pulse width on heart rate. Summary of the heart rate response to stimulation before versus after atropine administration (t_4 = 2.993, *P = 0.0402). mean ± s.e.m.; paired, two-tailed *t*-test.

Frequency (Hz)	Optical: Baseline heart rate (bpm)	Optical: Stimulation heart rate (bpm)	Optical: Δ Heart rate (%)	Electrical: Baseline heart rate (bpm)	Electrical: Stimulation heart rate (bpm)	Electrical: Δ Heart rate (%)
**Intact RVNS** (n = 8)						
1	560.7 ± 5.5	552.5 ± 5.9	-1.5 ± 0.2	594.1 ± 4.4	587.9 ± 4.5	-1.0 ± 0.2
5	561.6 ± 6.6	533.6 ± 5.3	-5.0 ± 0.3	593.4 ± 5.5	556.0 ± 8.7	-6.3 ± 0.8
10	552.0 ± 8.0	481.6 ± 8.7	-12.8 ± 0.4	590.7 ± 6.1	509.5 ± 6.3	-13.8 ± 0.6
20	558.3 ± 6.3	401.3 ± 13.0	-28.1 ± 2.2	591.6 ± 5.7	370.3 ± 32.0	-37.7 ± 5.0
**RVNx caudal RVNS** (n = 3)						
1	545.8 ± 12.9	535.9 ± 13.1	-1.8 ± 0.6	564.2 ± 8.9	555.3 ± 8.0	-1.6 ± 0.5
5	550.9 ± 11.5	501.5 ± 9.2	-9.0 ± 1.0	567.7 ± 7.9	519.9 ± 13.3	-8.4 ± 1.9
10	562.2 ± 8.2	459.6 ± 19.5	-18.3 ± 2.5	568.2 ± 4.6	449.2 ± 33.0	-21.0 ± 5.7
20	549.3 ± 11.5	302.9 ± 46.0	-44.9 ± 8.1	564.2 ± 7.4	276.2 ± 47.6	-51.0 ± 8.5
**BVNx caudal RVNS** (n = 3)						
1	530.0 ± 6.6	516.4 ± 5.9	-2.6 ± 0.4	558.9 ± 7.1	548.3 ± 10.6	-1.9 ± 0.7
5	534.0 ± 2.7	493.6 ± 5.7	-7.5 ± 1.5	549.8 ± 10.6	508.3 ± 28.5	-7.7 ± 3.4
10	560.6 ± 6.4	478.2 ± 16.7	-14.7 ± 2.3	546.9 ± 9.5	457.2 ± 33.8	-16.5 ± 5.1
20	545.6 ± 3.0	370.8 ± 44.5	-32.0 ± 8.3	551.5 ± 8.1	354.4 ± 69.3	-36.1 ± 11.5
**RVNx cranial RVNS** (n = 5)						
1	533.4 ± 10.6	531.2 ± 10.7	-0.4 ± 0.1	569.5 ± 13.0	568.2 ± 13.5	-0.2 ± 0.2
5	539.3 ± 11.0	536.9 ± 10.5	-0.4 ± 0.2	569.6 ± 14.1	559.0 ± 19.5	-2.0 ± 1.3
10	556.6 ± 12.9	556.0 ± 12.7	-0.1 ± 0.1*	576.1 ± 10.8	539.5 ± 18.8	-6.5 ± 1.8*
20	550.1 ± 12.1	549.3 ± 11.7	-0.1 ± 0.1**	576.1 ± 11.2	519.5 ± 17.3	-9.9 ± 1.8**
**BVNx cranial RVNS** (n = 5)						
1	518.5 ± 7.4	519.7 ± 8.5	0.2 ± 0.2	546.7 ± 7.5	546.9 ± 8.0	0.0 ± 0.2
5	526.1 ± 9.0	525.5 ± 9.4	-0.1 ± 0.1	545.2 ± 9.1	545.9 ± 8.9	0.1 ± 0.1
10	547.4 ± 5.9	545.5 ± 5.5	-0.3 ± 0.2	542.4 ± 8.5	539.7 ± 11.0	-0.5 ± 0.5
20	535.7 ± 6.9	534.9 ± 7.2	-0.2 ± 0.1**	545.6 ± 7.8	528.3 ± 8.7	-3.2 ± 0.4**

Supplemental Table 2. *In vivo* optogenetic versus electrical stimulation of the vagus nerve. Table corresponds to Figure 4. Frequency response curves summarizing the effects of optogenetic versus electrical stimulation of the right vagus nerve on heart rate in the intact state, of the caudal end following RVNx and BVNx, and of the cranial end following RVNx (t_4 = 3.576, *P = 0.0232 at 10 Hz; t_4 = 5.229, **P = 0.0064 at 20 Hz) and BVNx (t_4 = 8.588, **P = 0.0010 at 20 Hz). mean ± s.e.m.; paired, two-tailed t-test.

Frequency (Hz) (*n* = 7)	Baseline heart rate (bpm)	Stimulation heart rate (bpm)	Δ Heart rate (%)
1	464.8 ± 22.6	471.7 ± 22.1	1.5 ± 0.4
2	458.6 ± 21.3	476.2 ± 22.6	3.8 ± 0.6
5	459.6 ± 22.1	486.5 ± 20.6	6.1 ± 1.1
10	455.7 ± 20.8	499.5 ± 20.4	9.9 ± 1.8
20	462.4 ± 20.2	499.8 ± 18.3	8.3 ± 1.1
**Pulse width (ms)** (*n* = 6)			
1	469.5 ± 37.5	482.9 ± 33.8	3.4 ± 1.6
2	475.0 ± 38.8	492.0 ± 35.3	4.1 ± 1.8
5	475.3 ± 36.3	500.4 ± 33.0	5.9 ± 2.4
10	464.2 ± 32.0	499.0 ± 31.2	7.9 ± 2.2
**RSG versus RT2G** (*n* = 7)			
RSG	461.7 ± 24.2	504.1 ± 23.1	9.5 ± 1.8**
RT2G	486.1 ± 39.4	487.6 ± 39.3	0.3 ± 0.2
**Propranolol** (*n* = 4)			
Pre	471.5 ± 23.4	498.0 ± 26.0	5.6 ± 1.4*
Post	364.1 ± 20.1	364.7 ± 20.5	0.1 ± 0.1

Supplemental Table 3. *In vivo* optogenetic stimulation of noradrenergic neurons in the RSG. Table corresponds to Figure 6. Dose response curves summarizing the effects of altering frequency and pulse width on heart rate. Summary of the heart rate response to stimulation of the RSG versus RT2G (t_6 = 5.435, ***P* = 0.0016). Summary of the heart rate response to RSG stimulation before versus after propranolol administration (t_3 = 3.951, **P* = 0.0289). mean ± s.e.m.; paired, two-tailed *t*-test.

Why is mixed recruitment of efferents/afferents by electrical stimulation more effective at rapidly increasing HR compared to optical pacing that is designed to only stimulate a pure population of nerves? Fig 4c - Given the relative delay in the optical versus electrical stimulation response, I wonder what is the evidence that the slower HR change with optical stimulation is not due to incomplete expression of the optically activated channel or a phased or delayed pattern of recruitment?

We speculate that the more rapid decrease in heart rate with electrical compared to optogenetic stimulation is due to: 1) non-orderly fiber recruitment (as suggested by the reviewer) and 2) recruitment of both efferent and afferent fibers. In normal physiology, motor units are recruited in order of size, from small to large, with increasing levels of motor activation[60,61]. Electrical stimulation results in a non-physiological, non-orderly recruitment of motor units, activating large myelinated fibers before small myelinated fibers[62-65]. In contrast, Llewellyn *et al*. demonstrated that optogenetic stimulation results in physiological, orderly recruitment in the sciatic nerve (motor nerve that innervates the gastrocnemius muscle) of Thy1-ChR2-YFP mice[62]. Similar to our data, the authors observed longer latency from the initiation of nerve stimulation to depolarization on the gastrocnemius electromyogram and time required for contraction and relaxation of the gastrocnemius muscle with optogenetic compared to electrical stimulation. In addition to non-orderly fiber recruitment, electrical stimulation activates both efferent and afferent fibers in the vagus nerve of ChAT-ChR2-eYFP mice, whereas optogenetics stimulation only activates efferent fibers. Our data shows that electrical stimulation of afferent fibers enhances efferent parasympathetic outflow and decreases heart rate following ipsilateral and bilateral vagotomy (Figure 4g and h, red line). Therefore, we believe that the heart rate response with electrical

stimulation of the intact vagus nerve (Figure 4c) is a cumulative response from activation of both efferent and afferent fibers, each of which is contributing to a reduction in heart rate.

ChAT is a transferase enzyme required for the synthesis of acetylcholine, the primary neurotransmitter released by all cholinergic neurons. ChAT was used as a promoter to drive the expression of ChR2-eYFP in cholinergic neurons in ChAT-ChR2-eYFP mice. In these mice, we observed robust expression of ChR2-eYFP in the cervical vagus nerve (Figure 4b), and therefore, do not believe that incomplete ChR2-eYFP expression was a limitation.

How do your results unravel the previous findings re electrical stimulation of the PV ganglia (i.e. bradycardia followed by tachycardia), as mentioned in the Discussion?

Immunohistochemical studies have shown that cardiac ganglia such as those that are clustered around the pulmonary veins are a heterogeneous population, which includes parasympathetic, sympathetic, and sensory neurons[66,67]. Previous functional studies have shown that electrical stimulation of pulmonary vein ganglia result in a biphasic heart rate response of bradycardia followed by tachycardia[10,68], likely due to activation of both parasympathetic and sympathetic neurons contained in the ganglia. In contrast to electrical stimulation, which lacks specificity, optogenetics allows for precise control of defined cell populations. Our data show the heart rate response with pure stimulation of cholinergic neurons in the IPV-GP—bradycardia without tachycardia—and highlight the importance of using precise tools to dissect cardiac neural circuitry. Future studies will use optogenetics to characterize the cardiovascular response to stimulation of different subpopulations of cardiac neurons including sensory neurons.

The post vagotomy experiments with a different response to optical (none) and electrical (HR slowing) is fascinating. Nevertheless, I wonder how you can be sure it is not due to lack of adequate expression/capture/recruitment using the optical method? For example, an electrode recording showing an impulse with optical stimulation in the rostral limb of the severed vagus would strengthen your conclusion that the biology, rather than technical aspects of your approach, was driving your findings.

As discussed above, ChAT is a transferase enzyme required for the synthesis of acetylcholine, the primary neurotransmitter released by all cholinergic neurons. ChAT was used as a promoter to drive the expression of ChR2-eYFP in cholinergic neurons in ChAT-ChR2-eYFP mice. In these mice, we observed robust expression of ChR2-eYFP in the cervical vagus nerve (Figure 4b), and therefore, do not believe that ChR2-eYFP expression was a limitation. The mouse cervical vagus nerve is approximately 100 µm in diameter[69] and blue light attenuation is approximately 56% at a depth of 100 µm[70]. In our vagus nerve stimulation experiments, the average laser power for optogenetic stimulation was 77 ± 6 mW. At a depth of 100 µm, a substantial laser power of 33 mW was still delivered. Thus, we do not believe that fiber capture was a limitation either.

The functional readout for our vagus nerve stimulation experiments was heart rate. In each ChAT-ChR2-eYFP mouse, a significant decrease in heart rate was observed with electrical and optogenetic stimulation of the intact vagus nerve (Figure 4d). In the same animals, a change in heart rate was no longer observed with optogenetic stimulation following transection of the vagus nerve below the optical fiber (cranial/rostral vagus nerve stimulation) (Figure 4g and h, blue line). We have no reason to believe that the optogenetic stimulation was unable to capture or recruit fibers following transection in the same animals. Indeed, in a separate subset of animals, a significant decrease in heart rate was observed with optogenetic stimulation following transection of the vagus nerve above the optical fiber (caudal vagus nerve stimulation) (Figure 4e and f, blue line). Together, these data suggest that our findings are driven by biology and that the transection

did not affect the ability of the optogenetic stimulation to capture or recruit fibers due to nerve injury or other potential mechanisms.

Recording compound action potentials from the mouse vagus nerve is technically challenging[71]. Even if we were able to obtain compound action potential recordings, we would expect to see no impulses with optogenetic stimulation of the cranial/rostral end of the vagus nerve following transection, since there is presumably no expression of ChR2-eYFP in the vagal afferent fibers.

Paragraph 2, page 10 - The contrast between findings on sympathetic neurons in canines (post ganglionic neurons mostly in the middle cervical ganglia of the PV chain) versus mice (showing a craniomedial localization) might be due to species differences.

We agree with the reviewer that there may be differences in neuronal organization across species. Interestingly, in small mammals (e.g., mouse, rats, guniea pigs) cardiac ganglia are located on the dorsal atrial wall[11,12], whereas in large mammals (e.g., pigs, dogs, humans) cardiac ganglia are located in atrial and ventricular epicardial fat pads[72-74]. In future studies, we are planning on applying techniques described in this manuscript to larger animal models to characterize interspecies differences in cardiac innervation.

Minor a minimum of 3 beats is excessively vague, arbitrary and, perhaps, insufficient to represent actual rates (i.e. 3 is a small number).

We apologize for the vagueness of this statement. For heart rate, the average heart rate over a half a second was taken at baseline and during stimulation. For the P wave duration, 3 beats were measured at baseline and 3 beats were measured during simulation. This has been revised in the manuscript.

Reviewer #3 (Remarks to the Author):

In this well-written manuscript the authors present a novel tool for studying the role of parasympathetic and sympathetic nervous system in murine cardiac electrophysiology using optogenetics. They use parasympathetic and sympathetic specific Cre lines in combination with AAV driven fluorescent proteins to visualize the cardiac autonomic nervous system. Moreover, using tissue-specific ChR2 expression they specifically stimulate either the parasympathetic or sympathetic nervous system and investigated the effect on heart rate. The authors conclude to have provided novel insight into neural regulation of heart rate and tools for studying neuronal cardiac circuits or neural control of other organs.

We thank the reviewer for careful reading of the manuscript and providing constructive comments. We have addressed the reviewer's comments point-by-point below.

comments

The 3D visualizations of the neural circuit anatomy are beautiful but passive (movies showing pre-defined angles and magnifications), it would be great if the authors could provide files where the user can decide at what angle and magnification the 3D reconstructions are visualized. Is this possible? 3D pdf for example?

All data from this manuscript including imaging data will be uploaded to the Stimulating Peripheral Activity to Relieve Conditions (SPARC) data repository and be made available to the public upon approval from the journal.

In addition, we have setup our own webpage with an embedded volume viewer to visualize and manipulate some iDISCO-cleared hearts. The website address is:

https://www.ics.uci.edu/~fowlkes/SPARC/volume/

We would also be glad to share entire raw imaging datasets of whole iDISCO-cleared hearts with the reviewer. However, these datasets are large (~100 gb)

Figure 2 and labeling of the cholinergic neurons. This part is confusing. Please explain the function of the human Synapsin I promotor. What is the reason for using the two-component expression system? Please elaborate.

Human Synapsin I (hSyn1) is localized to the nerve terminals of axons and plays a role in neurotransmitter release. The hSyn1 promoter limits expression to neurons and is a widely-used pan-neuronal promoter[16,75,76].

A two-component AAV system was used to control the labeling density[16]. The two-component system consists of an expression vector (e.g., ssAAV-PHP.S:TRE-DIO-XFPs in Figure 2) delivered at a high dose and an inducer vector (ssAAV-PHP.S:ihSyn1-DIO-tTA) delivered at a variable dose. The expression of the gene of interest (e.g., fluorescent proteins (XFPs)) in a cell is dependent on co-transduction by the inducer vector. By varying the dose of the inducer vector, gene expression can be titrated. With the dense labeling (Figure 2a), anatomical tracing of nerve fibers is challenging because of the close apposition of fibers in nerve bundles. However, with the two-component expression system, we were able to reduce the fraction of labeled cells and trace individual fibers (Figure 2b and c). More specific details about the two-component system are available in prior publications by our group[16,75].

Why not placing Cre directly under control of the Synapsin I promotor? Figure 2c shows results of an entirely different expression system. Please explain.

We used Cre-dependent AAVs and ChAT-IRES-Cre transgenic mice to restrict the expression of the fluorescent reporter genes to cholinergic neurons as we were interested in tracing this subpopulation of cardiac neurons. If Cre was placed under the control of the Synapsin I promoter (i.e., Syn1-Cre), the fluorescent reporter genes would be expressed in all neurons and not just cholinergic neurons.

How did the authors verify that labeling was indeed restricted to the cholinergic neurons? Low levels of Cre expression outside these neurons would generate aspecific labeling. Please provide additional data verifying specificity using an independent marker.

We performed additional immunohistochemistry to address the reviewer's comment. Please see Response Figure 3 and Supplemental Figure 3. In ChAT-IRES-Cre mice injected with ssAAV-PHP.S:CAG-DIO-eYFP, GFP expression was highly specific for cholinergic neurons in cardiac ganglia ($100.0 \pm 0.0\%$ of GFP+ neurons were ChAT+) (Supplemental Figure 3d and e).

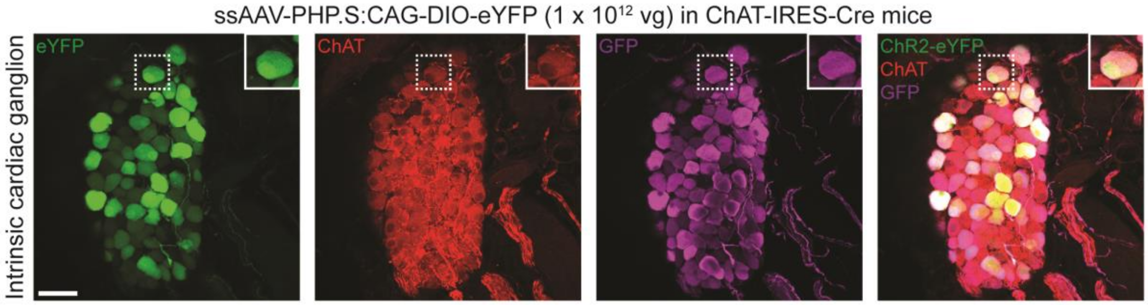

Response Figure 3. ssAAV-PHP.S:CAG-DIO-eYFP was systemically administered to ChAT-IRES-Cre mice at 1 x 10^{12} vg per mouse. Three weeks later, eYFP fluorescence was assessed. Representative single-plane confocal images of a cardiac ganglion with native eYFP signal (green) and ChAT (red) and GFP (magenta) staining. Scale bar is 50 µm.

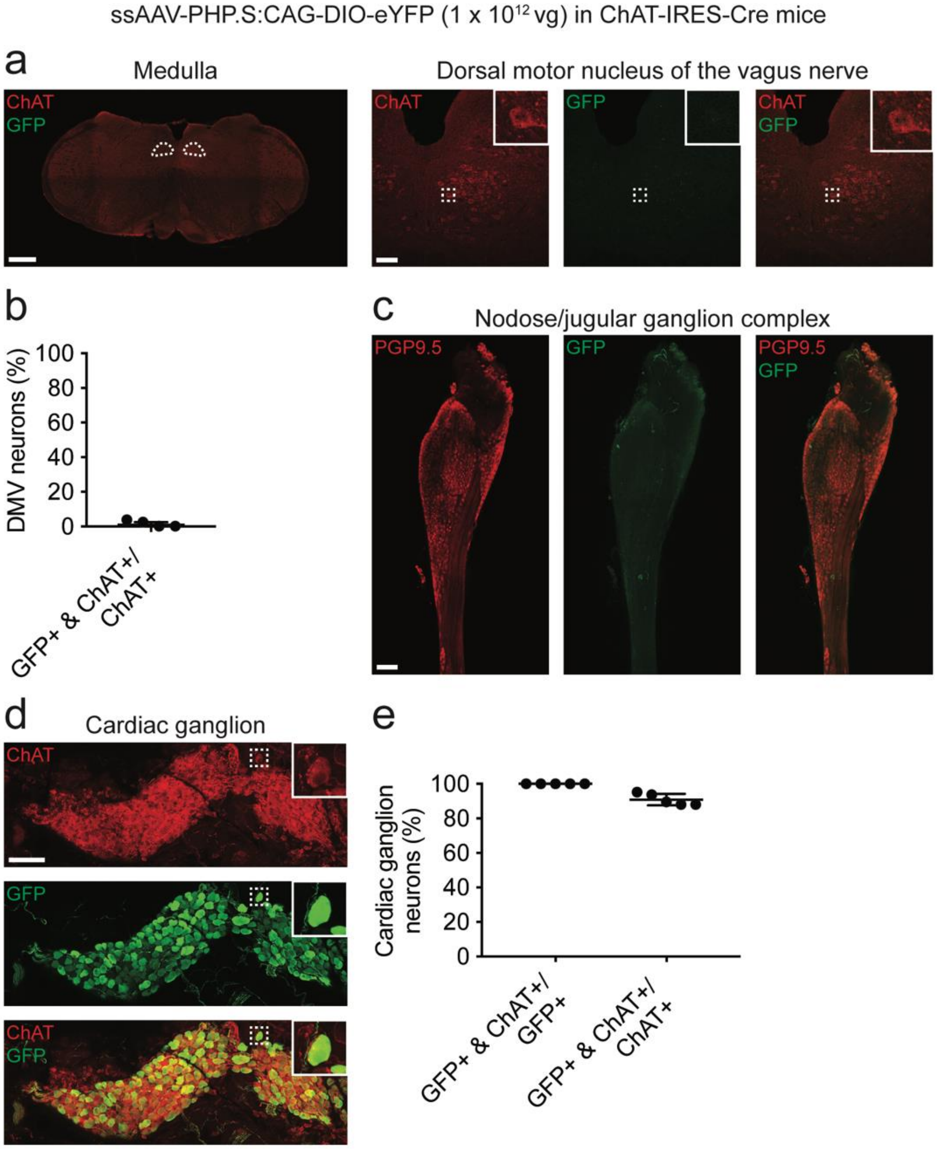

Supplemental Figure 3. AAV-PHP.S preferentially transduces peripheral versus central cholinergic neurons. ssAAV-PHP.S:CAG-DIO-eYFP was systemically administered to ChAT-IRES-Cre mice at 1 x 10^{12} vg per mouse. Three weeks later, eYFP fluorescence was assessed using an antibody for GFP. (**a**) Single-plane confocal images of the medulla (left) and dorsal motor nucleus of the vagus nerve (DMV) (right) whole-mount stained with ChAT (red) and GFP (green). White dashed ovals in the medulla show the location of the DMV. White dashed boxes in the DMV images indicate location of higher magnification images in white boxes. (**b**) Percentage of DMV neurons expressing GFP and ChAT over those expressing ChAT. (**c**) MIP images of the nodose/jugular ganglion complex whole-mount stained with PGP9.5 (red) and GFP (green). (**d**) MIP images of a cardiac ganglion from a heart whole-mount stained with PGP9.5 (red) and GFP (green). White dashed boxes indicate location of higher magnification images in white boxes. (**e**) Percentage of cardiac ganglion neurons expressing GFP and ChAT over those expressing GFP or ChAT, indicating specificity or efficiency of viral transduction, respectively. *n* = 4 mice (**b**) and

5 mice (**e**); mean ± s.e.m.. Scale bars are 500 µm (**a** (left)), 100 µm (**a** (right), **c**, **d** (right), and 1 mm (**d** (left)).

The authors use multiple fluorescent proteins to visualize the parasympathetic nervous system. Please explain why.

Most, if not all, commercially available antibodies against ChAT recognize a central rather than peripheral isoform of ChAT, labeling peripheral cholinergic neurons weakly[18]. An antibody has been developed against the peripheral isoform of ChAT[19]; however, it is not yet commercially available. Therefore, we used antibody against ChAT in the medulla in the central nervous system and an antibody against PGP9.5 as a surrogate for ChAT in cardiac ganglia in the peripheral nervous system due to the weak labeling and high background associated with ChAT staining in the periphery. We performed additional immunohistochemistry that shows strong colocalization of GFP with ChAT in ChAT-IRES-Cre mice injected with ssAAV-PHP.S:CAG-DIO-eYFP. These data are included in the above response and in Supplemental Figure 3.

Figure 2D. It is unclear how the sections are oriented. I suggest to add an overview section indicating the location of the sections shown. Moreover, Hcn4 is used to mark the Sinus node and AV node. To me, however, the Hcn4 staining seems stronger outside the areas marked with the dashed white lines than inside. The AV node does not seem to be labeled. Please address this issue.

A cartoon depicting orientation of the section has been added to Figure 2c. HCN4 is the best available marker to identify the SA node and AV node. While HCN channels are expressed predominately within the cardiac conduction system (SA node, AV node, and His bundle), they are also expressed to a lesser extent outside of the conduction system in atrial and ventricular myocardial tissue[77]. Therefore, we relied primarily on anatomical landmarks (i.e. sulcus terminalis and superior vena cava for the SA node; base of the right atrium and interatrial septum for AV node), in addition to HCN4 immunostaining, to identify the SA node and AV node.

Page 5. The authors use ChR2-enhanced yellow fluorescent protein but switch to GFP in their descriptions, which is confusing.

We apologize for the confusion. A widely-used antibody against GFP (Aves Labs, GFP-1020; 545 citations on CiteAb) was utilized to amplify the detection of endogenous ChR2-eYFP signals. For all quantifications, GFP expression was used.

“rostral” is archaic, cranial or anterior is more commonly used.

We now use cranial rather than rostral in the manuscript.

Figure 4 C. The authors compare optical stimulation of the right vagal nerve with electrical stimulation of the right vagal nerve. The approaches differ in 2 ways. 1) Only a part of the RVNS is stimulated optically whereas the whole RVNS is stimulated during electrical stimulation, and 2) optical stimulation in itself differs from electrical stimulation. Why didn’t the authors compare electrical stimulation with optical stimulation of both the efferent and afferent fibers in the vagal nerve? This allows for discriminating the different responses to the different approaches.

We thank the reviewer for this insightful question. To the best of our knowledge, pan-neuronal Cre knock-in mouse lines have not been well characterized in the peripheral nervous system. Therefore, we used a validated ChAT-IRES-Cre line[78,79] for our optogenetic studies. As better

transgenic mouse models are generated these studies will be expanded. Furthermore, we are currently developing intersectional tools to deliver genes of interest (e.g., fluorescent reporter proteins, opsins) to heart-specific efferent and afferent fibers of the vagus. Future studies will focus on optogenetic stimulation of heart-specific vagal efferents and afferents.

The authors report p wave fractionation during IPV-GP stimulation. Do the authors mean a biphasic p wave? It is known that the right and left atrium activation with a delay of about 5 ms. The activation wave propagates from the right atrium towards the left atrium via a dorsal myocardial connection. Do the authors think this delay between right and left atrium is increased during IPV-GP stimulation? Or does IPV-GP stimulation affect atrial conduction?

In some instances, a biphasic P wave was noted, which as the reviewer mentions, reflects impulse propagation from the right to left atria. However, we noted increases in P wave duration as well as changes in morphology during IPV-GP stimulation (Response Figure 8). The prolongation of P wave duration does suggest that increased time is required to depolarize the atria with optogenetic stimulation of the IPV-GP. Fractionated atrial electrograms are thought to indicate conduction slowing or block and sites for autonomic innervation and are thought to reflect the structural and functional properties of the atrial myocardium[80,81]. Indeed, in human patients undergoing atrial fibrillation ablation, our group has previously shown that pharmacologic stimulation of the cardiac parasympathetic nerves causes fractionation of atrial electrograms during sinus rhythm[82]. The contribution of autonomic nervous system to fractionation of atrial electrograms has also been shown in a canine model[83]. We are not able to state whether the fractionation in the atrial electrograms were due to slowed conduction or altered conduction pathways.

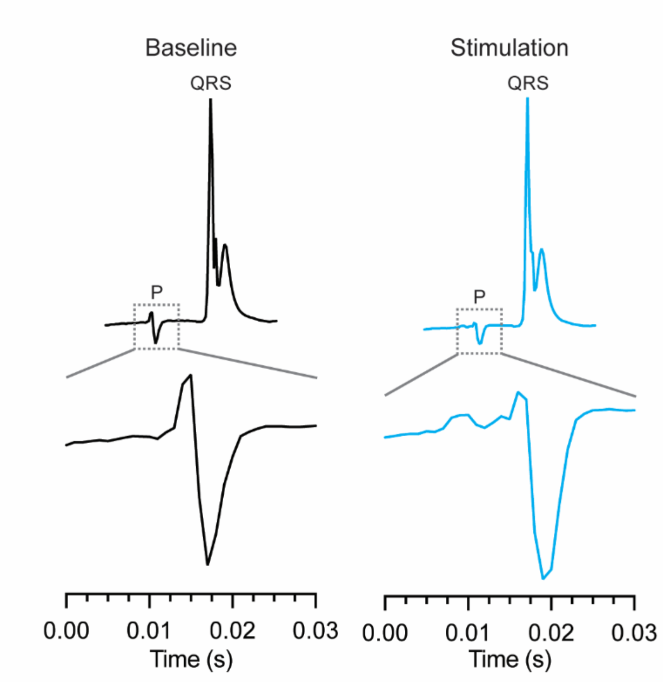

Response Figure 8. Example of a cardiac electrogram at baseline and during stimulation of the IPV-GP. Note in both cases the P waves (shown in the gray dashed boxes) were biphasic but during stimulation the P wave was longer in duration and fractionated.

Page 5 line 31. The authors mention that the nerve fiber passes through the AV node but does not synapse. Where is the nerve going? Was ventricular conduction affected?

While synapses were not visualized in our study, it is possible that fibers from the IPV-GP pass through the AV node region and synapse on neighboring atrial or ventricular myocardium.

The focus of our manuscript was on identifying cholinergic and noradrenergic neurons involved in heart rate regulation and not in control of ventricular electrophysiology. However, in a subset of ChAT-ChR2-eYFP mice, we performed dual optical mapping of membrane voltage (V_m) and intracellular Ca^{2+} transients (CaT) of the heart during optogenetic stimulation of the IPV-GP (Response Figure 9a). We observed atrial and ventricular changes in V_m and CaT activation maps during stimulation (Response Figure 9b) and a trend towards an increase in the V_m and CaT delay at the atria and base of the heart (Response Figure 9c and d). Future studies should focus on cholinergic and noradrenergic control of ventricular electrophysiology.

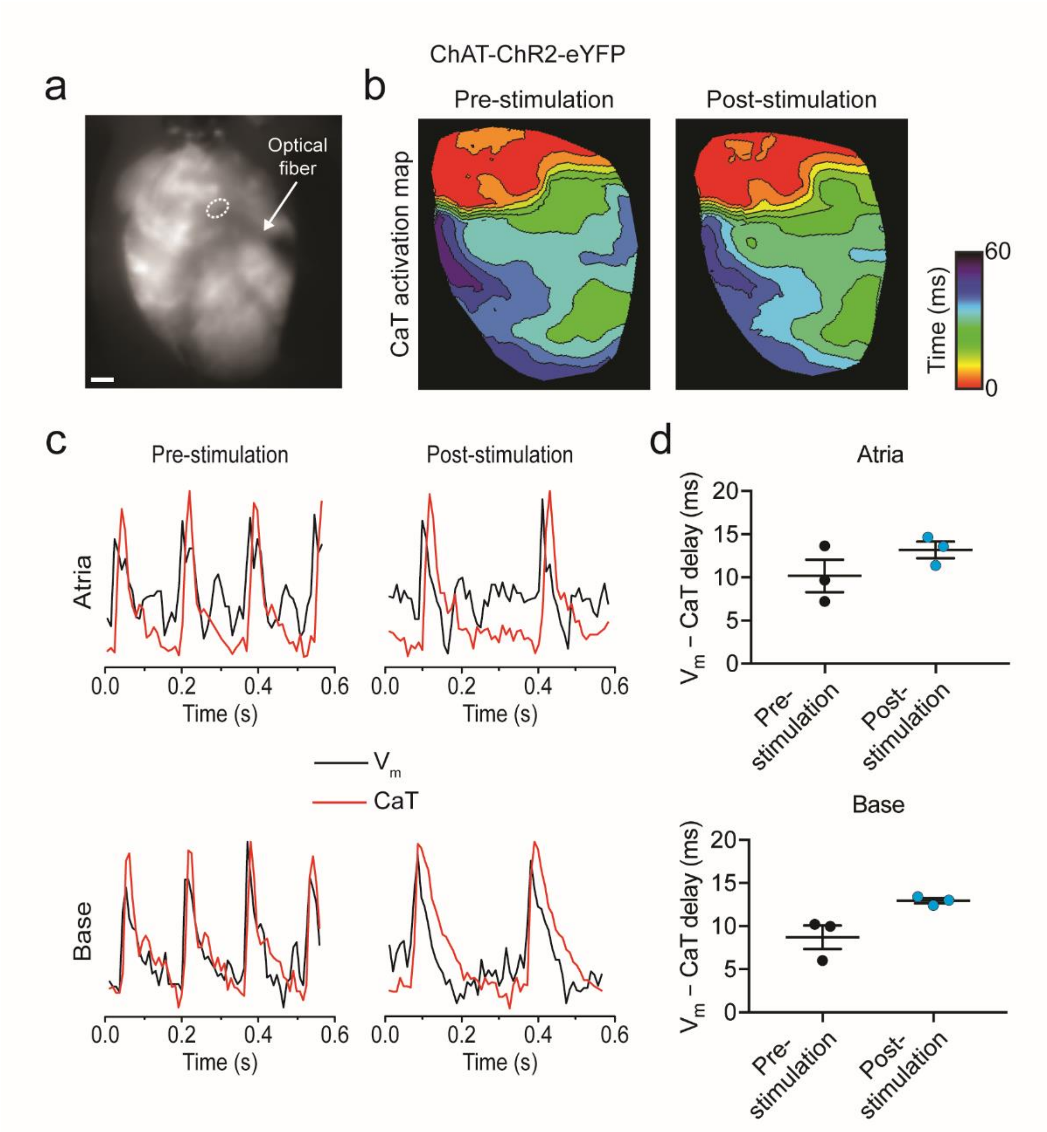

Response Figure 9. Optical mapping of *ex vivo* heart during optogenetic stimulation of cholinergic neurons in the IPV-GP. (**a**) Image of the dorsal side of a ChAT-ChR2-eYFP mouse

heart showing the position of a laser-coupled optical fiber used for focal illumination of the IPV-GP (circle). (**b**) Representative isochronal calcium transient (CaT) activation maps before and immediately after stimulation. (**c**) Representative membrane voltage (V_m) and CaT signals from the atria (top) and base of the heart (bottom) before (left) and immediately after stimulation (right). (**d**) Summary of the delay between V_m and CaT signals before versus immediately after stimulation in the atria ($t_2 = 2.980$, $P = 0.0965$) (top) and base of the heart ($t_2 = 3.022$, $P = 0.0943$) (bottom). $n = 3$ mice (**d**); mean ± s.e.m.; paired, two-tailed *t*-test. Scale bar is 1 mm (**a**).

On page 7 Figure 4G is discussed before Figure 4F. Please adjust the figure order to the text.

We have made this correction in the manuscript.

On page 8 and Figure 6, please provide validation of the specificity of the TH Cre driver.

The paravertebral ganglia including the stellate ganglia primarily contain the cell bodies of sympathetic postganglionic neurons. TH is the rate-limiting enzyme in the production of norepinephrine and is a widely-used marker of sympathetic neurons. We counted an average of 291 ± 62 stellate ganglion neurons from 6 TH-ChR2-eYFP mice. GFP expression was highly specific for TH+ neurons in the stellate ganglia (98.7 ± 0.4% of GFP+ neurons were TH+) and all TH+ neurons were GFP+ (100.0 ± 0.0%) (Figure 6c and Response Figure 10).

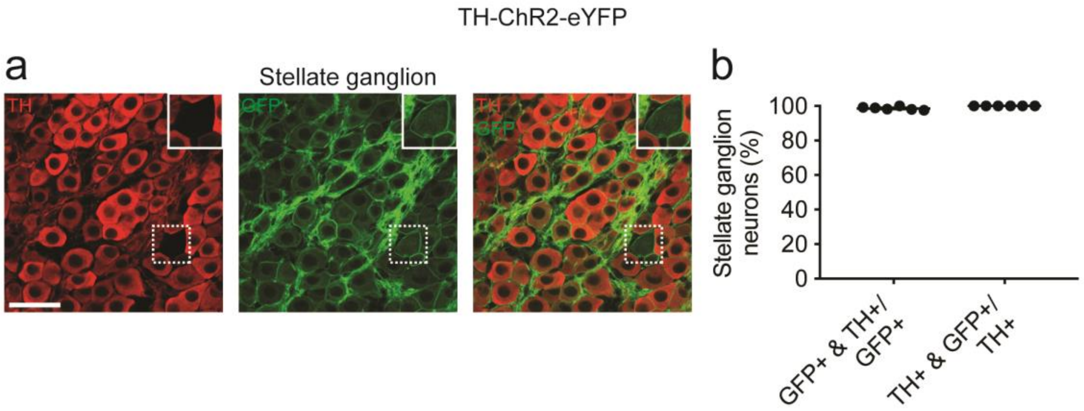

Response Figure 10. ChR2-EYFP expression in stellate ganglion of TH-ChR2-eYFP mice. (**a**) Representative single-plane confocal images of a stellate ganglion from a TH-ChR2-eYFP mouse whole-mount stained with TH (red) and GFP (green). Inset shows a neuron that is TH- and GFP+. (**b**) Percentage of stellate ganglion neurons expressing GFP and TH over those expressing GFP or TH. Scale bar is 50 µm. $n = 6$ mice (**b**).

Discussion conclusion 3, it is not clear how the authors arrive at this conclusion. Please better explain. “central mechanisms”?

Our functional data demonstrate that activation of vagal afferent fibers decreases heart rate (Figure 4g and h). Anatomical studies have shown that vagal afferent neurons synapse on neurons in the nucleus tractus solitarii (NTS) in the medulla in the brainstem[84]. Neurons in the NTS then project to vagal nuclei (nucleus ambiguus and dorsal motor nucleus) in the medulla to modulate parasympathetic efferent outflow[84] and to sympathetic nuclei (rostral ventrolateral medulla) in the medulla to modulate sympathetic efferent flow[85]. Consistent with previous studies

on the classic vasovagal reflex (Bezold-Jarish reflex)[86-88], we speculate that vagal afferents act on central nuclei to decrease heart rate by increasing parasympathetic efferent outflow and decreasing sympathetic efferent outflow. Further, the decrease in heart rate with electrical stimulation of vagal afferents following bilateral vagotomy (Figure 4h) is evidence that vagal afferent stimulation decreases sympathetic efferent outflow to the heart, since vagal efferent pathways were transected. Therefore, vagal afferent-mediated effects on heart rate must be relayed through the central nervous system.

References: In some cases the author list is abbreviated and in other case it is not. Please adjust to the correct style.

We used the *Nature* referencing style. In the *Nature* referencing style, all authors are included in reference lists unless there are six or more authors in which case only the first author is given, followed by 'et al.'.

REFERENCES

1 Renier, N. *et al.* iDISCO: a simple, rapid method to immunolabel large tissue samples for volume imaging. *Cell* **159**, 896-910, doi:10.1016/j.cell.2014.10.010 (2014).

2 Hama, H. *et al.* Scale: a chemical approach for fluorescence imaging and reconstruction of transparent mouse brain. *Nat Neurosci* **14**, 1481-1488, doi:10.1038/nn.2928 (2011).

3 Erturk, A. *et al.* Three-dimensional imaging of solvent-cleared organs using 3DISCO. *Nat Protoc* **7**, 1983-1995, doi:10.1038/nprot.2012.119 (2012).

4 Ke, M. T., Fujimoto, S. & Imai, T. SeeDB: a simple and morphology-preserving optical clearing agent for neuronal circuit reconstruction. *Nat Neurosci* **16**, 1154-1161, doi:10.1038/nn.3447 (2013).

5 Chung, K. *et al.* Structural and molecular interrogation of intact biological systems. *Nature* **497**, 332-337, doi:10.1038/nature12107 (2013).

6 Treweek, J. B. *et al.* Whole-body tissue stabilization and selective extractions via tissue-hydrogel hybrids for high-resolution intact circuit mapping and phenotyping. *Nat Protoc* **10**, 1860-1896, doi:10.1038/nprot.2015.122 (2015).

7 Susaki, E. A. *et al.* Whole-brain imaging with single-cell resolution using chemical cocktails and computational analysis. *Cell* **157**, 726-739, doi:10.1016/j.cell.2014.03.042 (2014).

8 Liebmann, T. *et al.* Three-Dimensional Study of Alzheimer's Disease Hallmarks Using the iDISCO Clearing Method. *Cell Rep* **16**, 1138-1152, doi:10.1016/j.celrep.2016.06.060 (2016).

9 Lagerweij, T. *et al.* Optical clearing and fluorescence deep-tissue imaging for 3D quantitative analysis of the brain tumor microenvironment. *Angiogenesis* **20**, 533-546, doi:10.1007/s10456-017-9565-6 (2017).

10 Zarzoso, M. *et al.* Nerves projecting from the intrinsic cardiac ganglia of the pulmonary veins modulate sinoatrial node pacemaker function. *Cardiovasc Res* **99**, 566-575, doi:10.1093/cvr/cvt081 (2013).

11 Rysevaite, K. *et al.* Immunohistochemical characterization of the intrinsic cardiac neural plexus in whole-mount mouse heart preparations. *Heart Rhythm* **8**, 731-738, doi:10.1016/j.hrthm.2011.01.013 (2011).

12 Rysevaite, K. *et al.* Morphologic pattern of the intrinsic ganglionated nerve plexus in mouse heart. *Heart Rhythm* **8**, 448-454, doi:10.1016/j.hrthm.2010.11.019 (2011).

13 Ng, G. A., Brack, K. E. & Coote, J. H. Effects of direct sympathetic and vagus nerve stimulation on the physiology of the whole heart--a novel model of isolated Langendorff perfused rabbit heart with intact dual autonomic innervation. *Exp Physiol* **86**, 319-329 (2001).

14 Brack, K. E., Coote, J. H. & Ng, G. A. Vagus nerve stimulation protects against ventricular fibrillation independent of muscarinic receptor activation. *Cardiovasc Res* **91**, 437-446, doi:10.1093/cvr/cvr105 (2011).

15 Deverman, B. E. *et al.* Cre-dependent selection yields AAV variants for widespread gene transfer to the adult brain. *Nat Biotechnol* **34**, 204-209, doi:10.1038/nbt.3440 (2016).

16 Chan, K. Y. *et al.* Engineered AAVs for efficient noninvasive gene delivery to the central and peripheral nervous systems. *Nat Neurosci* **20**, 1172-1179, doi:10.1038/nn.4593 (2017).

17 Bedbrook, C. N., Deverman, B. E. & Gradinaru, V. Viral Strategies for Targeting the Central and Peripheral Nervous Systems. *Annu Rev Neurosci* **41**, 323-348, doi:10.1146/annurev-neuro-080317-062048 (2018).

18 Bellier, J. P. & Kimura, H. Peripheral type of choline acetyltransferase: biological and evolutionary implications for novel mechanisms in cholinergic system. *J Chem Neuroanat* **42**, 225-235, doi:10.1016/j.jchemneu.2011.02.005 (2011).

19 Tooyama, I. & Kimura, H. A protein encoded by an alternative splice variant of choline acetyltransferase mRNA is localized preferentially in peripheral nerve cells and fibers. *J Chem Neuroanat* **17**, 217-226 (2000).
20 Deng, W., Goldys, E. M., Farnham, M. M. & Pilowsky, P. M. Optogenetics, the intersection between physics and neuroscience: light stimulation of neurons in physiological conditions. *Am J Physiol Regul Integr Comp Physiol* **307**, R1292-1302, doi:10.1152/ajpregu.00072.2014 (2014).
21 Barber, R. P. *et al.* The morphology and distribution of neurons containing choline acetyltransferase in the adult rat spinal cord: an immunocytochemical study. *J Comp Neurol* **229**, 329-346, doi:10.1002/cne.902290305 (1984).
22 Coote, J. H. & Chauhan, R. A. The sympathetic innervation of the heart: Important new insights. *Auton Neurosci* **199**, 17-23, doi:10.1016/j.autneu.2016.08.014 (2016).
23 Buckley, U. *et al.* Bioelectronic neuromodulation of the paravertebral cardiac efferent sympathetic outflow and its effect on ventricular electrical indices. *Heart Rhythm* **14**, 1063-1070, doi:10.1016/j.hrthm.2017.02.020 (2017).
24 Buckley, U. *et al.* Targeted stellate decentralization: Implications for sympathetic control of ventricular electrophysiology. *Heart Rhythm* **13**, 282-288, doi:10.1016/j.hrthm.2015.08.022 (2016).
25 Ardell, J. L., Rajendran, P. S., Nier, H. A., KenKnight, B. H. & Armour, J. A. Central-peripheral neural network interactions evoked by vagus nerve stimulation: functional consequences on control of cardiac function. *Am J Physiol Heart Circ Physiol* **309**, H1740-1752, doi:10.1152/ajpheart.00557.2015 (2015).
26 Vaseghi, M. *et al.* Modulation of regional dispersion of repolarization and T-peak to T-end interval by the right and left stellate ganglia. *Am J Physiol Heart Circ Physiol* **305**, H1020-1030, doi:10.1152/ajpheart.00056.2013 (2013).
27 Yamakawa, K. *et al.* Electrophysiological effects of right and left vagal nerve stimulation on the ventricular myocardium. *Am J Physiol Heart Circ Physiol* **307**, H722-731, doi:10.1152/ajpheart.00279.2014 (2014).
28 Yamakawa, K. *et al.* Vagal nerve stimulation activates vagal afferent fibers that reduce cardiac efferent parasympathetic effects. *Am J Physiol Heart Circ Physiol* **309**, H1579-1590, doi:10.1152/ajpheart.00558.2015 (2015).
29 Wengrowski, A. M. *et al.* Optogenetic release of norepinephrine from cardiac sympathetic neurons alters mechanical and electrical function. *Cardiovasc Res* **105**, 143-150, doi:10.1093/cvr/cvu258 (2015).
30 Yu, L. *et al.* Optogenetic Modulation of Cardiac Sympathetic Nerve Activity to Prevent Ventricular Arrhythmias. *J Am Coll Cardiol* **70**, 2778-2790, doi:10.1016/j.jacc.2017.09.1107 (2017).
31 Prando, V. *et al.* Dynamics of neuroeffector coupling at cardiac sympathetic synapses. *J Physiol* **596**, 2055-2075, doi:10.1113/JP275693 (2018).
32 Shi, W. *et al.* Distribution and prevalence of hyperpolarization-activated cation channel (HCN) mRNA expression in cardiac tissues. *Circulation research* **85**, e1-e6 (1999).
33 Milanesi, R., Baruscotti, M., Gnecchi-Ruscone, T. & DiFrancesco, D. Familial sinus bradycardia associated with a mutation in the cardiac pacemaker channel. *New England Journal of Medicine* **354**, 151-157 (2006).
34 Nof, E. *et al.* Point mutation in the HCN4 cardiac ion channel pore affecting synthesis, trafficking, and functional expression is associated with familial asymptomatic sinus bradycardia. *Circulation* **116**, 463-470 (2007).
35 Schulze-Bahr, E. *et al.* Pacemaker channel dysfunction in a patient with sinus node disease. *The Journal of clinical investigation* **111**, 1537-1545 (2003).

36 Ueda, K. *et al.* Functional characterization of a trafficking-defective HCN4 mutation, D553N, associated with cardiac arrhythmia. *Journal of Biological Chemistry* **279**, 27194-27198 (2004).

37 Herrmann, S., Layh, B. & Ludwig, A. Novel insights into the distribution of cardiac HCN channels: an expression study in the mouse heart. *Journal of molecular and cellular cardiology* **51**, 997-1006 (2011).

38 Moosmang, S. *et al.* Cellular expression and functional characterization of four hyperpolarization-activated pacemaker channels in cardiac and neuronal tissues. *European journal of biochemistry* **268**, 1646-1652 (2001).

39 Hoesl, E. *et al.* Tamoxifen-inducible gene deletion in the cardiac conduction system. *Journal of molecular and cellular cardiology* **45**, 62-69 (2008).

40 Stieber, J. *et al.* The hyperpolarization-activated channel HCN4 is required for the generation of pacemaker action potentials in the embryonic heart. *Proceedings of the National Academy of Sciences* **100**, 15235-15240 (2003).

41 Fenske, S. *et al.* Sick sinus syndrome in HCN1-deficient mice. *Circulation*, CIRCULATIONAHA. 113.003712 (2013).

42 Dobrzynski, H. *et al.* Site of origin and molecular substrate of atrioventricular junctional rhythm in the rabbit heart. *Circulation research* **93**, 1102-1110 (2003).

43 Yoo, S. *et al.* Localization of Na+ channel isoforms at the atrioventricular junction and atrioventricular node in the rat. *Circulation* **114**, 1360-1371 (2006).

44 Greener, I. *et al.* Molecular architecture of the human specialised atrioventricular conduction axis. *Journal of molecular and cellular cardiology* **50**, 642-651 (2011).

45 Acosta, C. *et al.* HCN1 and HCN2 in Rat DRG neurons: levels in nociceptors and non-nociceptors, NT3-dependence and influence of CFA-induced skin inflammation on HCN2 and NT3 expression. *PloS one* **7**, e50442 (2012).

46 Weng, X., Smith, T., Sathish, J. & Djouhri, L. Chronic inflammatory pain is associated with increased excitability and hyperpolarization-activated current (Ih) in C-but not Aδ-nociceptors. *PAIN®* **153**, 900-914 (2012).

47 Yao, H., Donnelly, D. F., Ma, C. & LaMotte, R. H. Upregulation of the hyperpolarization-activated cation current after chronic compression of the dorsal root ganglion. *Journal of Neuroscience* **23**, 2069-2074 (2003).

48 Papp, I., Holló, K. & Antal, M. Plasticity of hyperpolarization-activated and cyclic nucleotid-gated cation channel subunit 2 expression in the spinal dorsal horn in inflammatory pain. *European Journal of Neuroscience* **32**, 1193-1201 (2010).

49 Chaplan, S. R. *et al.* Neuronal hyperpolarization-activated pacemaker channels drive neuropathic pain. *Journal of Neuroscience* **23**, 1169-1178 (2003).

50 Jiang, Y.-Q. *et al.* Axonal accumulation of hyperpolarization-activated cyclic nucleotide-gated cation channels contributes to mechanical allodynia after peripheral nerve injury in rat. *PAIN®* **137**, 495-506 (2008).

51 Schnorr, S. *et al.* HCN2 channels account for mechanical (but not heat) hyperalgesia during long-standing inflammation. *PAIN®* **155**, 1079-1090 (2014).

52 Emery, E. C., Young, G. T., Berrocoso, E. M., Chen, L. & McNaughton, P. A. HCN2 ion channels play a central role in inflammatory and neuropathic pain. *Science* **333**, 1462-1466 (2011).

53 Thompson, R. J., Doran, J. F., Jackson, P., Dhillon, A. P. & Rode, J. PGP 9.5--a new marker for vertebrate neurons and neuroendocrine cells. *Brain Res* **278**, 224-228 (1983).

54 Crick, S. J. *et al.* Innervation of the human cardiac conduction system. A quantitative immunohistochemical and histochemical study. *Circulation* **89**, 1697-1708 (1994).

55 Horackova, M., Armour, J. A. & Byczko, Z. Distribution of intrinsic cardiac neurons in whole-mount guinea pig atria identified by multiple neurochemical coding. A confocal microscope study. *Cell Tissue Res* **297**, 409-421 (1999).

56 Crick, S. J., Sheppard, M. N., Ho, S. Y. & Anderson, R. H. Localisation and quantitation of autonomic innervation in the porcine heart I: conduction system. *J Anat* **195 (Pt 3)**, 341-357 (1999).

57 Horackova, M., Slavikova, J. & Byczko, Z. Postnatal development of the rat intrinsic cardiac nervous system: a confocal laser scanning microscopy study in whole-mount atria. *Tissue Cell* **32**, 377-388, doi:10.1054/tice.2000.0126 (2000).

58 Pauziene, N. *et al.* Innervation of the rabbit cardiac ventricles. *J Anat* **228**, 26-46, doi:10.1111/joa.12400 (2016).

59 Pauziene, N. *et al.* Neuroanatomy of the Pig Cardiac Ventricles. A Stereomicroscopic, Confocal and Electron Microscope Study. *Anat Rec (Hoboken)* **300**, 1756-1780, doi:10.1002/ar.23619 (2017).

60 Henneman, E. Relation between size of neurons and their susceptibility to discharge. *Science* **126**, 1345-1347 (1957).

61 Henneman, E., Somjen, G. & Carpenter, D. O. Functional Significance of Cell Size in Spinal Motoneurons. *J Neurophysiol* **28**, 560-580, doi:10.1152/jn.1965.28.3.560 (1965).

62 Llewellyn, M. E., Thompson, K. R., Deisseroth, K. & Delp, S. L. Orderly recruitment of motor units under optical control in vivo. *Nat Med* **16**, 1161-1165, doi:10.1038/nm.2228 (2010).

63 Singh, K., Richmond, F. J. & Loeb, G. E. Recruitment properties of intramuscular and nerve-trunk stimulating electrodes. *IEEE Trans Rehabil Eng* **8**, 276-285 (2000).

64 Fang, Z. P. & Mortimer, J. T. Selective activation of small motor axons by quasi-trapezoidal current pulses. *IEEE Trans Biomed Eng* **38**, 168-174, doi:10.1109/10.76383 (1991).

65 Lertmanorat, Z. & Durand, D. M. Extracellular voltage profile for reversing the recruitment order of peripheral nerve stimulation: a simulation study. *J Neural Eng* **1**, 202-211, doi:10.1088/1741-2560/1/4/003 (2004).

66 Hoard, J. L. *et al.* Cholinergic neurons of mouse intrinsic cardiac ganglia contain noradrenergic enzymes, norepinephrine transporters, and the neurotrophin receptors tropomyosin-related kinase A and p75. *Neuroscience* **156**, 129-142, doi:10.1016/j.neuroscience.2008.06.063 (2008).

67 Hoover, D. B. *et al.* Localization of multiple neurotransmitters in surgically derived specimens of human atrial ganglia. *Neuroscience* **164**, 1170-1179, doi:10.1016/j.neuroscience.2009.09.001 (2009).

68 Allen, E. *et al.* Electrophysiological effects of nicotinic and electrical stimulation of intrinsic cardiac ganglia in the absence of extrinsic autonomic nerves in the rabbit heart. *Heart Rhythm* **15**, 1698-1707, doi:10.1016/j.hrthm.2018.05.018 (2018).

69 Falcone, J. D. *et al.* A wrappable microwire electrode for awake, chronic interfacing with small diameter autonomic peripheral nerves. *bioRxiv*, doi:10.1101/402925 (2018).

70 Yizhar, O., Fenno, L. E., Davidson, T. J., Mogri, M. & Deisseroth, K. Optogenetics in neural systems. *Neuron* **71**, 9-34, doi:10.1016/j.neuron.2011.06.004 (2011).

71 Silverman, H. A. *et al.* Standardization of methods to record Vagus nerve activity in mice. *Bioelectronic Medicine* **4**, 3, doi:10.1186/s42234-018-0002-y (2018).

72 Arora, R. C., Waldmann, M., Hopkins, D. A. & Armour, J. A. Porcine intrinsic cardiac ganglia. *Anat Rec A Discov Mol Cell Evol Biol* **271**, 249-258, doi:10.1002/ar.a.10030 (2003).

73 Yuan, B. X., Ardell, J. L., Hopkins, D. A., Losier, A. M. & Armour, J. A. Gross and microscopic anatomy of the canine intrinsic cardiac nervous system. *Anat Rec* **239**, 75-87, doi:10.1002/ar.1092390109 (1994).

74 Armour, J. A., Murphy, D. A., Yuan, B. X., Macdonald, S. & Hopkins, D. A. Gross and microscopic anatomy of the human intrinsic cardiac nervous system. *Anat Rec* **247**, 289-298, doi:10.1002/(SICI)1097-0185(199702)247:2<289::AID-AR15>3.0.CO;2-L (1997).

75 Challis, R. C. *et al.* Systemic AAV vectors for widespread and targeted gene delivery in rodents. *Nat Protoc* **14**, 379-414, doi:10.1038/s41596-018-0097-3 (2019).
76 Kugler, S., Kilic, E. & Bahr, M. Human synapsin 1 gene promoter confers highly neuron-specific long-term transgene expression from an adenoviral vector in the adult rat brain depending on the transduced area. *Gene Ther* **10**, 337-347, doi:10.1038/sj.gt.3301905 (2003).
77 Kurian, T., Ambrosi, C., Hucker, W., Fedorov, V. V. & Efimov, I. R. Anatomy and electrophysiology of the human AV node. *Pacing Clin Electrophysiol* **33**, 754-762, doi:10.1111/j.1540-8159.2010.02699.x (2010).
78 Chang, R. B., Strochlic, D. E., Williams, E. K., Umans, B. D. & Liberles, S. D. Vagal Sensory Neuron Subtypes that Differentially Control Breathing. *Cell* **161**, 622-633, doi:10.1016/j.cell.2015.03.022 (2015).
79 Hedrick, T. *et al.* Characterization of Channelrhodopsin and Archaerhodopsin in Cholinergic Neurons of Cre-Lox Transgenic Mice. *PLoS One* **11**, e0156596, doi:10.1371/journal.pone.0156596 (2016).
80 Chang, S. h. *et al.* Time-and frequency-domain characteristics of atrial electrograms during sinus rhythm and atrial fibrillation. *Journal of cardiovascular electrophysiology* **22**, 851-857 (2011).
81 Latchamsetty, R. & Morady, F. (Am Heart Assoc, 2011).
82 Lellouche, N. *et al.* Functional characterization of atrial electrograms in sinus rhythm delineates sites of parasympathetic innervation in patients with paroxysmal atrial fibrillation. *Journal of the American College of Cardiology* **50**, 1324-1331 (2007).
83 Lin, J. *et al.* Autonomic mechanism to explain complex fractionated atrial electrograms (CFAE). *Journal of cardiovascular electrophysiology* **18**, 1197-1205 (2007).
84 Standish, A., Enquist, L. W., Escardo, J. A. & Schwaber, J. S. Central neuronal circuit innervating the rat heart defined by transneuronal transport of pseudorabies virus. *J Neurosci* **15**, 1998-2012 (1995).
85 Strack, A. M., Sawyer, W. B., Hughes, J. H., Platt, K. B. & Loewy, A. D. A general pattern of CNS innervation of the sympathetic outflow demonstrated by transneuronal pseudorabies viral infections. *Brain Res* **491**, 156-162 (1989).
86 Mark, A. L. The Bezold-Jarisch reflex revisited: clinical implications of inhibitory reflexes originating in the heart. *J Am Coll Cardiol* **1**, 90-102 (1983).
87 Aviado, D. M. & Guevara Aviado, D. The Bezold-Jarisch reflex. A historical perspective of cardiopulmonary reflexes. *Ann N Y Acad Sci* **940**, 48-58 (2001).
88 Chapleau, M. W. & Sabharwal, R. Methods of assessing vagus nerve activity and reflexes. *Heart Fail Rev* **16**, 109-127, doi:10.1007/s10741-010-9174-6 (2011).

REVIEWERS' COMMENTS:

Reviewer #1 (Remarks to the Author):

The authors are commended for their comprehensive response to the review. The manuscript has significantly improved. One last issue is that the relevant work of Moreno and colleagues has been published in Frontiers and must be referenced in the main text: Moreno A, Endicott K, Skancke M, Dwyer MK, Brennan J, Efimov IR, Trachiotis G, Mendelowitz D, Kay MW. Sudden Heart Rate Reduction Upon Optogenetic Release of Acetylcholine From Cardiac Parasympathetic Neurons in Perfused Hearts. Front Physiol. 2019;10:1–11.

Reviewer #2 (Remarks to the Author):

congratulations

Reviewer #3 (Remarks to the Author):

The authors have addressed my comments satisfactorily.
A minor issue that could be taken into account in future studies: Hcn4 is expressed in the AV node and SAN, and is used in Figure 2 to detect these tissues. However, Hcn4 expression is not detected in the AV node in Figure 2c whereas a lot of background signal is visible. This method of AV node and SAN detection could be optimized or replaced.

REVIEWERS' COMMENTS:

We thank the editor and the reviewers for their thoughtful review of the manuscript again and for the opportunity to revise our work.

Reviewer #1 (Remarks to the Author):

The authors are commended for their comprehensive response to the review. The manuscript has significantly improved. One last issue is that the relevant work of Moreno and colleagues has been published in Frontiers and must be referenced in the main text: Moreno A, Endicott K, Skancke M, Dwyer MK, Brennan J, Efimov IR, Trachiotis G, Mendelowitz D, Kay MW. Sudden Heart Rate Reduction Upon Optogenetic Release of Acetylcholine From Cardiac Parasympathetic Neurons in Perfused Hearts. Front Physiol. 2019;10:1–11.

We thank the reviewer for careful reading of the manuscript again. We have added the important reference by Moreno and colleagues to the Introduction and Discussion sections.

Reviewer #2 (Remarks to the Author):

congratulations

We thank the reviewer for thorough reading of the manuscript again.

Reviewer #3 (Remarks to the Author):

The authors have addressed my comments satisfactorily.

A minor issue that could be taken into account in future studies: Hcn4 is expressed in the AV node and SAN, and is used in Figure 2 to detect these tissues. However, Hcn4 expression is not detected in the AV node in Figure 2c whereas a lot of background signal is visible. This method of AV node and SAN detection could be optimized or replaced.

We thank the reviewer for careful reading of the manuscript again and bringing to our attention the issue of HCN4 immunostaining of the SA and AV node. We have tried a number of antibodies against HCN4 at various concentrations in order to improve the signal and reduce the background. Unfortunately, this is a limitation of the currently available antibodies against HCN4. Thus, we relied on anatomical landmarks in the manuscript, in addition to HCN4 immunostaining, to identify the SA and AV node. As better antibodies become available in the future, HCN4 staining will be optimized.
